# Brain multi-omic Mendelian randomisation to identify novel drug targets for gliomagenesis

**DOI:** 10.1093/hmg/ddae168

**Published:** 2024-11-20

**Authors:** Zak A Thornton, Lily J Andrews, Huiling Zhao, Jie Zheng, Lavinia Paternoster, Jamie W Robinson, Kathreena M Kurian

**Affiliations:** MRC Integrative Epidemiology Unit (IEU), Bristol Medical School, University of Bristol, Oakfield House, Oakfield Grove, Bristol, BS8 2BN, United Kingdom; Population Health Sciences, Bristol Medical School, University of Bristol, Oakfield House, Oakfield Grove, Bristol, BS8 2BN, United Kingdom; Cancer Research Integrative Cancer Epidemiology Programme (ICEP), University of Bristol, Oakfield House, Oakfield Grove, Bristol, BS8 2BN, United Kingdom; Leeds Institute of Cardiovascular and Musculoskeletal Medicine, Faculty of Medicine and Health, University of Leeds, Leeds and NIHR Leeds Biomedical Research Centre, Leeds Teaching Hospitals NHS Trust, Chapel Allerton Hospital, Chapeltown Road, Leeds, LS7 4SA, United Kingdom; MRC Integrative Epidemiology Unit (IEU), Bristol Medical School, University of Bristol, Oakfield House, Oakfield Grove, Bristol, BS8 2BN, United Kingdom; Population Health Sciences, Bristol Medical School, University of Bristol, Oakfield House, Oakfield Grove, Bristol, BS8 2BN, United Kingdom; Cancer Research Integrative Cancer Epidemiology Programme (ICEP), University of Bristol, Oakfield House, Oakfield Grove, Bristol, BS8 2BN, United Kingdom; MRC Integrative Epidemiology Unit (IEU), Bristol Medical School, University of Bristol, Oakfield House, Oakfield Grove, Bristol, BS8 2BN, United Kingdom; MRC Integrative Epidemiology Unit (IEU), Bristol Medical School, University of Bristol, Oakfield House, Oakfield Grove, Bristol, BS8 2BN, United Kingdom; Department of Endocrine and Metabolic Diseases, Shanghai Institute of Endocrine and Metabolic Diseases, Ruijin Hospital, Shanghai Jiao Tong University School of Medicine, South Chongqing Road, Shanghai, 200025, China; Shanghai National Clinical Research Centre for Metabolic Diseases, Key Laboratory for Endocrine and Metabolic Diseases of the National Health Commission of the PR China, Shanghai Key Laboratory for Endocrine Tumor, State Key Laboratory of Medical Genomics, Ruijin Hospital, Shanghai Jiao Tong University School of Medicine, South Chongqing Road, Shanghai, 200025, China; MRC Integrative Epidemiology Unit (IEU), Bristol Medical School, University of Bristol, Oakfield House, Oakfield Grove, Bristol, BS8 2BN, United Kingdom; Population Health Sciences, Bristol Medical School, University of Bristol, Oakfield House, Oakfield Grove, Bristol, BS8 2BN, United Kingdom; NIHR Bristol Biomedical Research Centre, University Hospitals Bristol and Weston NHS Foundation Trust and University of Bristol, Oakfield House, Oakfield Grove, Bristol, BS8 2BN, United Kingdom; MRC Integrative Epidemiology Unit (IEU), Bristol Medical School, University of Bristol, Oakfield House, Oakfield Grove, Bristol, BS8 2BN, United Kingdom; MRC Integrative Epidemiology Unit (IEU), Bristol Medical School, University of Bristol, Oakfield House, Oakfield Grove, Bristol, BS8 2BN, United Kingdom; Population Health Sciences, Bristol Medical School, University of Bristol, Oakfield House, Oakfield Grove, Bristol, BS8 2BN, United Kingdom; Cancer Research Integrative Cancer Epidemiology Programme (ICEP), University of Bristol, Oakfield House, Oakfield Grove, Bristol, BS8 2BN, United Kingdom; Brain Tumour Research Centre, Bristol Medical School, University of Bristol, Department of Neuropathology, Lime Walk Buidling, Southmead Hospital, North Bristol NHS Trust, Bristol, BS10 5NB, United Kingdom

**Keywords:** glioma, Mendelian randomisation, quantitative trait loci, molecular traits, e/s/pQTL

## Abstract

**Background:**

Genetic variants associated with molecular traits that are also associated with liability to glioma can provide causal evidence for the identification and prioritisation of drug targets.

**Methods:**

We performed comprehensive two-sample Mendelian randomisation (Wald ratio and/or IVW) and colocalisation analyses of molecular traits on glioma. Instrumentable traits (QTLs *P* < 5 × 10^−8^) were identified amongst 11 985 gene expression measures, 13 285 splicing isoforms and 10 198 protein abundance measures, derived from 15 brain regions. Glioma summary-level data was extracted from a genome-wide association meta-analysis of 12 496 cases and 18 190 controls.

**Results:**

We found evidence for causal effect of 22 molecular traits (across 18 genes/proteins) on glioma risk. Thirteen molecular traits have been previously linked with glioma risk and five were novel; *HBEGF* (5q31.3) expression and all glioma [OR 1.36 (95%CI 1.19–1.55); *P* = 4.41 × 10^−6^]; a *CEP192* (18p11.21) splice isoform and glioblastoma [OR 4.40 (95%CI 2.28–8.48); *P* = 9.78 × 10^−4^]; a *FAIM* (3q22.3) splice isoform and all glioma [OR 2.72–3.43; *P* = 1.03 × 10^−5^ to 1.09 × 10^−5^]; a *SLC8A1* (2p22.1) splice isoform and all glioma [OR 0.37 (95%CI 0.24–0.56; *P* = 5.72 × 10^−6^]; D2HGDH (2q37.3) protein and all glioma [OR 0.86 (95%CI 0.80–0.92); *P* = 5.94 × 10^−6^)].

**Conclusions:**

We provide robust causal evidence for prioritising genes and their protein products in glioma research. Our results highlight the importance of alternative splicing as a mechanism in gliomagenesis and as an avenue for exploration of drug targets.

## Introduction

Glioma is the most common (~80%) primary malignant brain and central nervous system (CNS) tumour group, with age-standardised incidence rates ranging from 4.67 to 5.73 per 100 000 [1, 2]. Malignant brain tumours are responsible for the greatest years of potential life lost of all cancers (~20 years), with a comparatively shorter 5-year relative survival rate (34.9%) compared to other malignancies [3, 4]. Current standard-of-care guidelines include surgery, radiotherapy and chemotherapy (temozolomide), and as such there is an urgent need for new targeted treatments [5].

To date, previous studies have identified 27 genetic loci associated with glioma risk, implicating a total of approximately 50 genes; however, further analysis is required to determine both which genes these genetic variants map to, and whether such variation is causally implicated in gliomagenesis, or simply an observational association [6–11]. Furthermore, genotyping of large cohorts has been performed to link disease-associated genetic variants to gene regulatory mechanisms to improve understanding of disease aetiology [12]. Such genetic variants associated with these molecular traits are known as quantitative trait loci (QTL), and examples of these include measuring relative gene expression levels (eQTLs), splicing variation (sQTLs) and protein abundance (pQTLs). Integrating multi-dimensional omics data can further our understanding of the aetiology of complex traits by providing functional and mechanistic insight into these layered molecular relationships [13–15].

Application of Mendelian randomisation (MR) analysis to screen for causal molecular traits, is a useful tool to identify causal genes and pathways that can in turn be considered as potential drug targets. Data is available through the MetaBrain consortium, GTEx and BrainQTL that, in combination with the available glioma genome-wide association study (GWAS) data, enables the evaluation of gene expression levels, splicing variation and protein abundance across up to 15 brain regions as potential causal factors for glioma [16, 17].

In this study, we leveraged this multi-omic data derived in bulk brain regions in a combined MR-colocalisation framework, with the aim to identify robust causal evidence for aetiologically important genes for gliomagenesis.

## Results

### Estimating the causal effects of molecular mechanisms on glioma risk

Of the 10 488 genes for which expression (in one of five brain regions) could be instrumented, 10 006 (95.4%) had the exact, or proxy variants available in the glioma GWAS and therefore MR could be performed. Thirteen genes showed evidence for a causal effect of expression on at least one glioma outcome, across one or more brain region (and passed colocalisation and Steiger filter criteria, 34 significant results in total) after correction for multiple testing using Bonferroni’s method (*P* < 5.00 × 10^−6^) (Supplementary Fig. S1).

Of the 6200 genes for which alternative splicing could be instrumented (in one of 13 brain regions), 4496 (72.5%) had the exact, or proxy variants available in the glioma GWAS and therefore MR could be performed. Six genes showed evidence for a causal effect of splicing on at least one glioma outcome and across one or more brain region (and passed colocalisation and Steiger filtering criteria, 23 significant results in total) following correcting for multiple testing using Bonferroni’s method (*P* < 1.11 × 10^−5^) (Supplementary Fig. S2).

Of the 2191 proteins that could be instrumented, 1929 (88.0%) had the exact, or proxy variants available in the glioma GWAS and therefore MR could be performed. Three proteins showed evidence for a causal effect of cerebral cortex protein abundance on one or more glioma outcomes (and passed colocalisation and Steiger filtering criteria, five significant results in total) after correction for multiple testing using Bonferroni’s method (*P* < 2.59 × 10^−5^) (Supplementary Fig. S3).

**Figure 1:**
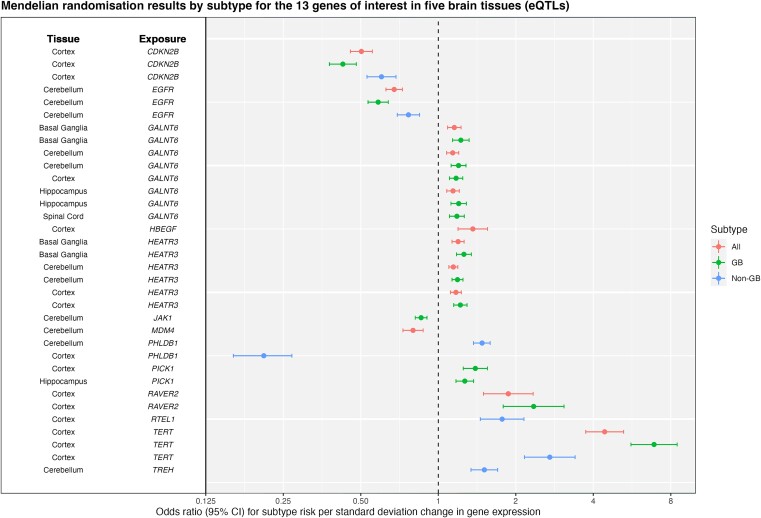
Forest plot showing Mendelian randomisation results by subtype (all glioma, GB and non-GB) for the 13 genes of interest in five brain tissues in the gene expression analysis. Results have strong evidence of colocalization (H_4_ > = 80%) and correct Steiger directionality.

**Figure 2:**
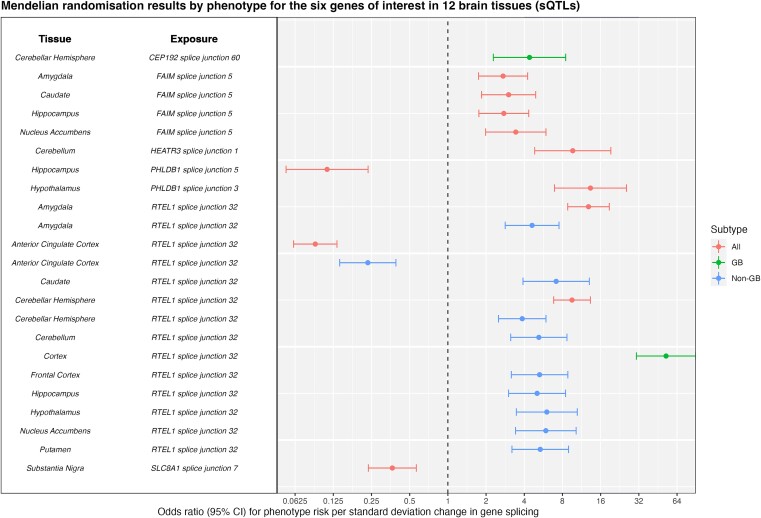
Forest plot showing Mendelian randomisation results by subtype (all glioma, GB and non-GB) for the six genes of interest in 12 brain tissues in the gene-splicing analysis. Results have strong evidence of colocalization (H_4_ > = 80%) and correct Steiger directionality.

**Figure 3:**
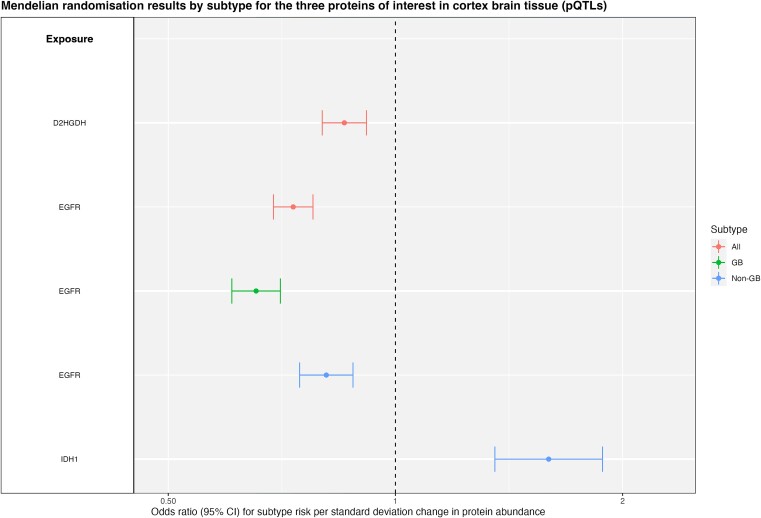
Forest plot showing Mendelian randomisation results by subtype (all glioma, GB and non-GB) for the three proteins of interest in cortex tissue in the protein abundance analysis. Results have strong evidence of colocalization (H_4_ > = 80%) and correct Steiger directionality.

For each analysis, a tissue-by-tissue breakdown are available in Supplementary Tables S1–S3. Results which passed sensitivity analyses, i.e. evidence of colocalisation (H_4_ ≥ 80%) and passed Steiger filtering are presented for eQTL, sQTL and pQTL in Tables 1–3 and Figs 1–3, respectively. Overall, 22 molecular traits (across 18 genes/proteins) met these criteria (referred to hereafter as ‘robust’ causal evidence). Results for all sensitivity analyses can be found in Supplementary Tables S4–S9.

**Table 1:** Results of the MR analysis investigating the effects of genetically proxied gene expression levels on genetic liability to glioma subtype.

**Tissue**	**Gene**	**Subtype**	**Method**	**No. of SNPs**	***P* Value**	**Odds Ratio (95% CI)**	**H4 (%)**	**Steiger Direction**	**Steiger *P* Value**	**Steiger Flag**
Cortex	*CDKN2B*	All	Wald ratio	1	7.17E-43	0.50 (0.46–0.55)	93	True	2.32E-19	Pass
Cortex	*CDKN2B*	GB	Wald ratio	1	6.22E-44	0.43 (0.38–0.48)	97	True	3.19E-15	Pass
Cortex	*CDKN2B*	Non-GB	Wald ratio	1	1.59E-14	0.60 (0.53–0.68)	93	True	2.91E-21	Pass
Cerebellum	*EGFR*	All	Wald ratio	1	1.07E-25	0.67 (0.63–0.73)	96	True	2.16E-10	Pass
Cerebellum	*EGFR*	GB	Wald ratio	1	2.89E-31	0.58 (0.53–0.64)	97	True	4.29E-09	Pass
Cerebellum	*EGFR*	Non-GB	Wald ratio	1	1.60E-07	0.77 (0.69–0.85)	98	True	2.65E-11	Pass
Basal Ganglia	*GALNT6*	All	Wald ratio	1	3.14E-06	1.15 (1.09–1.23)	99	True	4.78E-09	Pass
Basal Ganglia	*GALNT6*	GB	Wald ratio	1	9.08E-08	1.22 (1.14–1.32)	100	True	1.23E-08	Pass
Cerebellum	*GALNT6*	All	Wald ratio	1	3.14E-06	1.14 (1.08–1.20)	100	True	7.94E-25	Pass
Cerebellum	*GALNT6*	GB	Wald ratio	1	9.08E-08	1.20 (1.12–1.28)	100	True	1.76E-23	Pass
Cortex	*GALNT6*	GB	IVW	2	1.53E-07	1.17 (1.11–1.24)	100	True	6.87E-130	Pass
Hippocampus	*GALNT6*	All	Wald ratio	1	4.17E-06	1.14 (1.08–1.21)	99	True	5.29E-08	Pass
Hippocampus	*GALNT6*	GB	Wald ratio	1	2.15E-07	1.20 (1.12–1.29)	100	True	1.09E-07	Pass
Spinal Cord	*GALNT6*	GB	Wald ratio	1	8.40E-07	1.18 (1.11–1.26)	99	True	6.88E-07	Pass
Cortex	*HBEGF*	All	Wald ratio	1	4.41E-06	1.36 (1.19–1.55)	98	True	8.18E-17	Pass
Basal Ganglia	*HEATR3*	All	Wald ratio	1	1.50E-10	1.19 (1.13–1.26)	93	True	7.61E-11	Pass
Basal Ganglia	*HEATR3*	GB	Wald ratio	1	1.55E-11	1.26 (1.18–1.34)	95	True	2.58E-11	Pass
Cerebellum	*HEATR3*	All	Wald ratio	1	3.93E-11	1.14 (1.10–1.19)	96	True	6.45E-54	Pass
Cerebellum	*HEATR3*	GB	Wald ratio	1	6.74E-12	1.19 (1.13–1.25)	97	True	1.25E-51	Pass
Cortex	*HEATR3*	All	Wald ratio	1	1.95E-10	1.17 (1.12–1.23)	97	True	1.15E-139	Pass
Cortex	*HEATR3*	GB	Wald ratio	1	8.20E-11	1.22 (1.15–1.29)	97	True	3.76E-125	Pass
Cerebellum	*JAK1*	GB	Wald ratio	1	9.28E-09	0.86 (0.81–0.90)	89	True	2.68E-38	Pass
Cerebellum	*MDM4*	All	Wald ratio	1	9.54E-07	0.80 (0.73–0.87)	91	True	2.10E-08	Pass
Cerebellum	*PHLDB1*	Non-GB	Wald ratio	1	7.77E-25	1.48 (1.37–1.59)	97	True	3.48E-15	Pass
Cortex	*PHLDB1*	Non-GB	Wald ratio	1	1.02E-32	0.21 (0.16–0.27)	98	True	3.08E-02	Pass
Cortex	*PICK1*	GB	Wald ratio	1	1.92E-09	1.39 (1.25–1.55)	91	True	2.14E-31	Pass
Hippocampus	*PICK1*	GB	Wald ratio	1	3.95E-09	1.27 (1.17–1.37)	81	True	1.65E-06	Pass
Cortex	*RAVER2*	All	Wald ratio	1	3.19E-08	1.87 (1.50–2.34)	94	True	8.11E-06	Pass
Cortex	*RAVER2*	GB	Wald ratio	1	7.92E-10	2.35 (1.79–3.08)	98	True	1.42E-04	Pass
Cortex	*RTEL1*	Non-GB	Wald ratio	1	9.78E-09	1.77 (1.46–2.15)	94	True	1.13E-08	Pass
Cortex	*TERT*	All	Wald ratio	1	2.32E-66	4.43 (3.74–5.25)	100	True	3.55E-03	Pass
Cortex	*TERT*	GB	Wald ratio	1	6.92E-75	6.89 (5.60–8.47)	100	True	1.18E-01	Uncertain
Cortex	*TERT*	Non-GB	Wald ratio	1	6.69E-18	2.71 (2.16–3.40)	100	True	3.26E-05	Pass
Cerebellum	*TREH*	Non-GB	Wald ratio	1	1.59E-11	1.51 (1.34–1.70)	84	True	9.87E-07	Pass

Results presented are those which were deemed the most robust, i.e. met the Bonferroni-correct *P*-value threshold (*P* = 5.00 × 10^−6^) for the MR analysis, had strong evidence of colocalisation (H4 > 80%) and correctly orientated Steiger filtering direction.

**Table 2:** Results of the MR analysis investigating the effects of genetically proxied gene splicing levels on genetic liability to glioma subtype.

**Tissue**	**Gene (Splice Junction)**	**Subtype**	**Method**	**No. of SNPs**	***P* Value**	**Odds Ratio (95% CI)**	**H4 (%)**	**Steiger Direction**	**Steiger *P* Value**	**Steiger Flag**
Cerebellar Hemisphere	*CEP192 Splice Junction 60*	GB	Wald ratio	1	9.78E-06	4.40 (2.28–8.48)	90	True	7.79E-01	Uncertain
Amygdala	*FAIM Splice Junction 5*	All	Wald ratio	1	1.03E-05	2.72 (1.74–4.25)	94	True	6.91E-01	Uncertain
Caudate	*FAIM Splice Junction 5*	All	Wald ratio	1	1.09E-05	3.01 (1.84–4.92)	95	True	6.83E-01	Uncertain
Hippocampus	*FAIM Splice Junction 5*	All	Wald ratio	1	1.09E-05	2.76 (1.76–4.34)	94	True	6.64E-01	Uncertain
Nucleus Accumbens	*FAIM Splice Junction 5*	All	Wald ratio	1	1.03E-05	3.43 (1.98–5.93)	95	True	7.29E-01	Uncertain
Cerebellum	*HEATR3 Splice Junction 1*	All	Wald ratio	1	1.29E-10	9.64 (4.83–19.24)	91	True	9.38E-01	Uncertain
Hippocampus	*PHLDB1 Splice Junction 5*	All	Wald ratio	1	7.83E-09	0.11 (0.05–0.24)	87	True	9.29E-01	Uncertain
Hypothalamus	*PHLDB1 Splice Junction 3*	All	Wald ratio	1	8.40E-15	13.29 (6.92–25.55)	99	True	9.94E-01	Uncertain
Amygdala	*RTEL1 Splice Junction 32*	All	Wald ratio	1	4.38E-40	12.83 (8.80–18.72)	99	True	9.68E-01	Uncertain
Amygdala	*RTEL1 Splice Junction 32*	Non-GB	Wald ratio	1	8.36E-10	4.61 (2.83–7.51)	98	True	7.34E-01	Uncertain
Anterior Cingulate Cortex	*RTEL1 Splice Junction 32*	All	Wald ratio	1	3.89E-33	0.09 (0.06–0.13)	94	True	9.46E-01	Uncertain
Anterior Cingulate Cortex	*RTEL1 Splice Junction 32*	Non-GB	Wald ratio	1	2.39E-08	0.23 (0.14–0.39)	92	True	7.24E-01	Uncertain
Caudate	*RTEL1 Splice Junction 32*	Non-GB	Wald ratio	1	1.56E-10	7.13 (3.91–13.02)	92	True	8.68E-01	Uncertain
Cerebellar Hemisphere	*RTEL1 Splice Junction 32*	All	Wald ratio	1	4.38E-40	9.52 (6.82–13.28)	85	True	8.87E-01	Uncertain
Cerebellar Hemisphere	*RTEL1 Splice Junction 32*	GB	Wald ratio	1	1.58E-45	19.1 (12.7–28.72)	90	True	7.79E-01	Uncertain
Cerebellar Hemisphere	*RTEL1 Splice Junction 32*	Non-GB	Wald ratio	1	8.36E-10	3.85 (2.51–5.93)	95	True	6.23E-01	Uncertain
Cerebellum	*RTEL1 Splice Junction 32*	Non-GB	Wald ratio	1	2.32E-10	5.21 (3.13–8.68)	99	True	7.43E-01	Uncertain
Cortex	*RTEL1 Splice Junction 32*	GB	Wald ratio	1	4.42E-47	52.42 (30.59–89.81)	99	True	6.97E-01	Uncertain
Frontal Cortex	*RTEL1 Splice Junction 32*	Non-GB	Wald ratio	1	2.00E-10	5.28 (3.16–8.82)	99	True	7.94E-01	Uncertain
Hippocampus	*RTEL1 Splice Junction 32*	Non-GB	Wald ratio	1	8.36E-10	5.05 (3.01–8.46)	96	True	8.08E-01	Uncertain
Hypothalamus	*RTEL1 Splice Junction 32*	Non-GB	Wald ratio	1	2.00E-10	6.02 (3.46–10.47)	99	True	8.12E-01	Uncertain
Nucleus Accumbens	*RTEL1 Splice Junction 32*	Non-GB	Wald ratio	1	2.32E-10	5.92 (3.41–10.25)	99	True	8.16E-01	Uncertain
Putamen	*RTEL1 Splice Junction 32*	Non-GB	Wald ratio	1	1.56E-10	5.35 (3.2–8.93)	98	True	7.66E-01	Uncertain
Substantia Nigra	*SLC8A1 Splice Junction 7*	All	Wald ratio	1	5.72E-06	0.37 (0.24–0.56)	96	True	7.01E-01	Uncertain

Results presented here are those which were deemed the most robust, i.e. met the Bonferroni-correct *P*-value threshold (*P* = 1.11 × 10^−5^) for the MR analysis, had strong evidence of colocalisation (H4 > 80%) and correctly orientated Steiger filtering direction.

**Table 3:** Results of the MR analysis investigating the effects of genetically proxied protein abundance levels on genetic liability to glioma subtype.

**Protein**	**Subtype**	**Method**	**No. of variants**	***P*-value**	**Odds Ratio (95% CI)**	**H4 (%)**	**Steiger Direction**	**Steiger *P*-value**	**Steiger Flag**
D2HGDH	All	Wald Ratio	1	5.94E-06	0.89 (0.80–0.92)	98	True	1.77E-42	Pass
EGFR	All	Wald Ratio	1	3.52E-24	0.73 (0.69–0.78)	99	True	7.34E-18	Pass
EGFR	GB	Wald Ratio	1	2.19E-19	0.65 (0.61–0.70)	99	True	6.06E-16	Pass
EGFR	Non-GB	Wald Ratio	1	3.82E-07	0.81 (0.75–0.88)	99	True	5.23E-19	Pass
IDH1	Non-GB	Wald Ratio	1	2.37E-08	1.60 (1.35–1.88)	95	True	4.59E-05	Pass

Results presented here are those which were deemed the most robust, i.e. met the Bonferroni-correct *P*-value threshold (*P* = 2.59 × 10^−5^) for the MR analysis, had strong evidence of colocalisation (H4 > 80%) and correctly orientated Steiger filtering direction.

### Novel genes

Across the multi-omic analyses, we found robust genetic evidence for five genes that have not been implicated with glioma risk in previous genetic studies: *CEP192* (Centrosomal protein of 192 kDa), D2HGDH (D-2-hydroxyglutarate dehydrogenase), *FAIM* (Fas apoptosis inhibitory module), *HBEGF* (Heparin binding epidermal growth factor) and *SLC8A1* (Solute carrier family 8 member A1). We compared the non-significant causal effects in the five novel genes to confirm whether the direction of effect was consistent across subtypes of glioma but did not reach the relevant Bonferroni-corrected *P*-value. Four of the novel results had robust causal effects in all glioma and showed no differences in magnitude or direction in the glioblastoma and non-glioblastoma subtype. Alternative splicing of *CEP192* had a significant robust causal effect in glioblastoma, but not in non-glioblastoma or all glioma, but with no difference in the direction of effect. This causal effect appears to drive the non-significant causal effect ratio found in all glioma (Table 4).

All molecular traits with robust causal results were tested across all tissue types using a more lenient Bonferroni-corrected *P*-value (*P* < 4.04 × 10^−4^), derived from the number of ad-hoc tests performed (0.05/123). *FAIM* showed overlap between gene expression and alternative splicing (cerebellum in all glioma, *P* = 6.93 × 10^−5^) and *D2HGDH* showed overlap between protein abundance and gene expression (cortex in all glioma, *P* = 8.70 × 10^−5^) (Supplementary Table S10).

### Specificity analysis

For the 22 molecular traits (across 18 genes/proteins) that had robust evidence for a causal effect on glioma, we performed follow-up ad hoc MR analyses to determine whether these molecular traits also had causal evidence with putative glioma risk factors identified in the literature; allergies, asthma, altered low-density lipoprotein cholesterol (LDLc) levels and alcohol consumption. We found that of the novel molecular traits with robust causal evidence, *D2HGDH* was suggestively associated with asthma, *FAIM* was suggestively associated with LDL cholesterol, and *SLC8A1* was suggestively associated with allergies and asthma (*P* < 0.05), suggesting that these molecular trait effects are not independent of reported risk factors. The trait that was associated with the most robust molecular traits was LDLc, which was suggestively associated with nine molecular traits (*CDKN2B, FAIM, GALNT6, HEATR3, IDH1, PHLDB1, PICK1, RTEL1* and *TREH*). After imposing a Bonferroni-corrected *P*-value (*P* < 1.71 × 10^−4^), derived from the number of tests performed (0.05/292), only four molecular traits were still associated with LDLc (*CDKN2B, GALNT6, PHLDB1* and *RTEL1*) (Supplementary Table S11).

We tested the respective genetic instruments of the novel results and *RTEL1* using a hypothesis-free PheWAS screen. *RTEL1* is linked with telomere length which has been previously implicated in observational and MR studies to have an association with glioma risk [18–20]. Additionally, the considerable number of brain regions in which we found a robust causal effect for *RTEL1* warranted further analyses. We used the IEU OpenGWAS project PheWAS tool and PhenoScanner v2 to conduct a broad search for phenotypes which were strongly associated with the lead variant(s) for *CEP192* (rs11661802), D2HGDH (rs71430382), *FAIM* (rs371073 and rs371074), *HBEGF* (rs4150197) and *RTEL1* (rs6062302, rs2297440, rs2315009, rs3208007 and rs2777941) at genome-wide significance (*P* < 5 × 10^−8^) [21, 22]. We found that *CEP192* variant rs11661802 was associated with a few anthropometric traits such as height and ankle spacing width. D2HGDH variant rs71430382 was associated with immune system related traits such as eosinophil cell count, asthma, eczema, anaphylaxis and respiratory disease. *FAIM* variant rs71430382 was associated with various traits including testosterone levels, mean platelet volume and HbA1c levels. We found that *HBEGF* variant rs4150197 was associated with anthropometric traits, and various image-derived phenotypes from diffuse MRI. We also found *RTEL1* variant rs6062302 was significantly associated with immune system related traits such as dermatitis, eczema, Crohn’s disease, inflammatory bowel disease and allergic rhinitis (Supplementary Table S12). These results give molecular insight into the role these molecular traits have on biological mechanisms e.g. alternative splicing of *FAIM* appears to have a large range of effects on a number of different traits, which would suggest it is a poor drug target.

### Differential expression in molecular traits

We searched for differential gene expression of *HBEGF and D2HGDH* in non-tumour and glioma samples from the Rembrandt and TCGA studies, using the GlioVis tool [23]. In the Rembrandt study, there were no significant differences (*P* < 0.05) between non-tumour samples (controls) and any of the available glioma subtypes in *HBEGF* expression. However, there was a significant difference in expression between GB and astrocytoma (mean log2 mRNA expression 7.16 versus 7.02, respectively; *P* = 3.10 × 10^−5^). There were significant differences in *D2HGDH* expression between non-tumour samples (controls) and all glioma subtypes (GB (*P* = 1.30 × 10^−6^), astrocytoma (*P* = 9.00 × 10^−7^), oligodendroglioma (*P* = 1.50 × 10^−7^), mixed glioma (*P* = 9.40 × 10^−3^) or unknown histology (*P* = 2.50 × 10^−9^)) (Supplementary Fig. S4A and B) [24].

In the TCGA study, there were significant differences in *HBEGF* and *D2HGDH* expression between GB and non-GB subtypes (astrocytoma (*P* = 3.50 × 10^−8^ and 4.50 × 10^−12^, respectively), oligoastrocytoma (*P* = 1.00 × 10^−8^ and 4.90 × 10^−12^, respectively) and oligodendroglioma (*P* = 1.90 × 10^−10^ and *P* = 1.40 × 10^−20^)). Supplementary Fig. S4C and D) [24, 25].

Additionally, we searched for differences in isoform expression of *CEP192, FAIM*, *RTEL1* and *SLC8A1* using the GTEx portal tool [17]. We found that *CEP192* isoform expression (ENST00000513183.1) was significantly higher in the heterozygous variant (CT) compared to wild type (CC) in the cerebellar hemisphere (*P* = 4.90 × 10^−15^), and *FAIM* isoform expression was significantly lower in both lead variants in four brain regions (*P* = 7.13 × 10^−11^ to 1.29 × 10^−27^). The isoform expression was lower between all wild-type and the heterozygous variants in the *RTEL1* lead variants (*P* = 6.64 × 10^−18^ to 4.23 × 10^−75^), and the cerebellum had the highest isoform (ENST00000425905.5) expression (3.17 TPM) followed by the cerebellar hemisphere (2.96 TPM), which were two brain regions in which we found a robust causal effect. Finally, we found that *SLC8A1* isoform expression (ENST00000406785.6) was significantly higher in the heterozygous variant (GT) compared to wild type (GG) in the substantia nigra (*P* = 7.95 × 10^−9^) [17].

### Single cell-specific expression patterns

Using the Human Protein Atlas (HPA) v23 data, we identified three molecular traits with robust causal evidence that appear to be expressed in specific cell types [26]. *GALNT6* expression was enhanced in oligodendrocytes (54.3 TPM), *FAIM* expression was enhanced in cone and rod photoreceptors (217.5 and 185.1 TPM, respectively) and *SLC8A1* was group enriched and specifically expressed in excitatory neurons (1224.1 TPM), inhibitory neurons (602.1 TPM), microglia (548.4 TPM) and oligodendrocyte precursor cells (706.5 TPM). The complete list of cell-specific gene expression transcript counts can be found in Supplementary Table S13.

### Annotation of drug tractability for robustly causal genes and proteins

We used OpenTargets and Drug Gene Interaction Database (DGIdb) to gather evidence of drug tractability for the 18 molecular traits with robust evidence [27–29]. We found that 5/10 (50%) of the molecular traits identified in the expression analysis (*EGFR, HBEGF, JAK1, MDM4* and *TERT*), and 1/3 (33%) of the molecular traits identified in the protein abundance analysis (EGFR) were categorised as being part of the ‘druggable genome’. None of the genes identified in the alternative splicing analysis were deemed druggable (Supplementary Table S14).

*EGFR* has a variety of associated drugs which have been previously used in phase IV clinical trials to treat several different cancers. These drugs are mainly small molecule inhibitors (such as afatanib, erlotinib and gefitinib) and antibodies (such as cetuximab, necitumumab and panitumumab). *JAK1* also has associated small molecule inhibitors which are tyrosine-protein kinase JAK1 inhibitors including abrocitinib, baricitinib and ruxolitinib, but have been primarily used in trials to treat autoimmune diseases such as atopic eczema, myelofibrosis and rheumatoid arthritis (Supplementary Tables S15 and S16). One novel result, *HBEGF,* had a known drug (KHK-2866), an anti-HBEGF antibody which was trialled for use in advanced solid tumours and ovarian cancer. However, the study was discontinued after 22 months due to patients experiencing reversible neuropsychiatric toxicity, although the aetiology was not understood (Supplementary Tables S15 and S16) [30]. From the gene ontology and molecular pathway analyses conducted using OpenTargets, only *EGFR* and *HBEGF* appeared to share a biological/molecular pathway (Supplementary Tables S17 and S18).

## Discussion

In this study, we used a combined MR-colocalisation framework to estimate the causal effect of genetically proxied gene expression, alternative splicing and protein abundance levels on glioma risk. We found robust evidence that causally implicated 22 molecular traits (across 18 genes/proteins) with glioma risk: 13 in gene expression, six in alternative splicing, and three in protein abundance. For three genes (*HEATR3, PHLDB1* and *RTEL1*) there were both expression and splicing causal effects on glioma risk, and for one gene (*EGFR*) there were both expression and protein abundance causal effects on glioma risk).

For 13 of the genes that we found robust causal evidence for here, the loci have been previously implicated in GWAS/TWAS of glioma risk [6, 7]. However, these previous studies did not establish causality of all these genes, as we have. Additionally, we identified five genes that not been implicated in glioma previously: *CEP192* (18p11.21), *D2HGDH* (2q37.3) *FAIM* (3q22.3), *HBEGF* (5q13.3) and *SLC8A1* (2p22.1).

*CEP192* plays a significant role in centrosome biogenesis, the formation and function of mitotic spindles, and is therefore essential for accurate chromosome segregation during cell division [31]. Centrosomal abnormalities, especially amplification, are prominent in cancer and have been observed in GB. For example, expression of *CENPJ*, *PCM1* and *CEP55*, which are key centromere proteins, has been observed to promote cell proliferation and migration, and may even modulate treatment response for cancer [32–34]. Specific to and organised by *CEP192* is a signalling cascade that underlies centrosome maturation and separation through CEP192 binding to Aurora kinase A (AURKA) and polo-like kinase 1 (PLK1) [35]. Both AURKA and PLK1 are serine/threonine-protein kinases, have been implicated in gliomagenesis, and are potential drug targets (with ongoing clinical trials) for many different cancers [36–38]. Inhibition of *AURKA* and *PLK1* is particularly attractive as it mediates phosphorylation, and hence can regulate functions of downstream substrates, including binding with CEP192 [37, 39]. In the context of our putatively causal evidence of increased expression of *CEP192* leading to increased GB risk, we postulate that inhibiting *CEP192* expression, or reducing the number of CEP192-formed complexes, may rescue aberrant centrosome maturation and separation, although more work would be required to confirm this biological mechanism. As an example, we suggest that inhibiting downstream effects of the *CEP192* signalling cascade would result in fewer binding sites for the gamma-tubulin ring complex (γ-TuRC), which plays a central role in shaping cell division and may lead to a chemotherapeutic effect [40]. Furthermore, specifically targeting the interaction between the CEP192 and AURKA proteins has been postulated recently in the literature, if such a protein–protein interaction can be targeted [41]. Our instrument for *CEP192* was rs11661802, an sQTL associated with an alternative 3′ splice event that gives rise to transcript ENST00000513183, a predicted protein-coding transcript without a defined coding sequence (CDS). Interestingly, this transcript is much shorter (703 bp versus 7960 bp for the canonical transcript) and so would result in a much smaller protein at roughly 26 kDa (whereas the predominant isoform is large at ~280 kDa) [35]. An area of further research is to elucidate the role of this transcript and its potential protein product in centrosome biology and potentially cancer.

**Table 4:** Causal effects across the three MR analyses in the novel genes/proteins in each glioma subtype (all glioma, GB and non-GB). The results in bold face highlight the effects that met the Bonferroni-corrected *P*-value. Note that the results across each glioma subtype have the same direction of effect, but did not meet the Bonferroni-corrected *P*-value.

**Gene/Protein**	**Tissue**	**Subtype**	**Odds Ratio (95% CI)**	***P*-value**
*CEP192*	Cerebellar Hemisphere	All Glioma	2.37 (1.38–4.06)	1.74E-03
		**GB**	**4.40 (2.28–8.48)**	**9.78E-06**
		Non-GB	1.08 (0.53–2.21)	8.29E-01
D2HGDH	Cortex	**All Glioma**	**0.86 (0.80–0.92)**	**5.94E-06**
		GB	0.89 (0.82–0.91)	5.99E-03
		Non-GB	0.83 (0.76–0.91)	3.66E-03
*FAIM*	Amygdala	**All Glioma**	**2.72 (1.74–4.25)**	**1.03E-05**
		GB	2.96 (1.71–5.11)	9.89E-05
		Non-GB	2.36 (1.31–4.24)	4.13E-03
*FAIM*	Caudate	**All Glioma**	**3.01 (1.84–4.92)**	**1.09E-05**
		GB	3.32 (1.82–6.06)	9.64E-05
		Non-GB	2.56 (1.34–4.90)	4.36E-03
*FAIM*	Hippocampus	**All Glioma**	**2.76 (1.76–4.34)**	**1.09E-05**
		GB	3.02 (1.73–5.25)	9.64E-05
		Non-GB	2.38 (1.31–4.32)	4.36E-03
*FAIM*	Nucleus Accumbens	**All Glioma**	**3.43 (1.98–5.93)**	**1.03E-05**
		GB	3.80 (1.94–7.44)	9.89E-05
		Non-GB	2.87 (1.40–5.92)	4.13E-03
*HBEGF*	Cortex	**All Glioma**	**1.36 (1.19–1.55)**	**4.41E-06**
		GB	1.45 (1.24–1.71)	5.53E-06
		Non-GB	1.23 (1.03–1.46)	2.01E-02
*SLC8A1*	Substantia Nigra	**All Glioma**	**0.37 (0.24–0.56)**	**5.72E-06**
		GB	0.39 (0.23–0.67)	5.67E-04
		Non-GB	0.34 (0.19–0.60)	1.81E-04

*D2HGDH* is a mitochondrial enzyme which is responsible for the oxidation of D-2-hydroxyglutarate (D-2-HG)—one enantiomer of 2-hydroxyglutarate (2-HG)—into 2-oxoglutarate (2-OG), which is a key mediator of the Krebs cycle [42]. Accumulation of 2-HG has been associated with the pathogenesis of multiple types of cancer such as diffuse large B-cell lymphoma and glioma, and 2-hydroxyglutaric aciduria (2-HGA) [43–45]. D-2-HGA is clinically characterised by developmental delay, epilepsy and dysmorphic features. There are two main type of D-2-HGA: type I is caused by loss-of-function mutations in D2HGDH, and type II is caused by gain-of-functions IDH1/2 (isocitrate dehydrogenase 1/2) mutations, which results in the reduction of 2-OG back to D-2-HG, and therefore both mechanisms lead to D-2-HG accumulation [46]. *IDH1* is commonly mutated in glioma. In the most recent WHO classification of brain tumours, the presence of *IDH1* mutations is used as a diagnostic marker within glioma (such as astrocytoma, *IDH* mutant and GB, *IDH* mutant) [47]. More recently, irregular D-2-HG accumulation has been identified as biomarker for glioma diagnosis and cancer progression, and D-2-HG dubbed an ‘oncometabolite’ [48]. Therefore, increased D2HGDH protein abundance may be protective against glioma by preventing the accumulation of D-2-HG, via catalytic conversion of D-2-HG to 2-OG.

*FAIM* is involved in pathways such as apoptosis, autophagy, and neurogenesis regulation. The role of FAIM in these pathways is driven by alternative splicing, whereby two transcript variants have been characterised and studied: FAIM short (FAIM-S), which is expressed ubiquitously, plays a role in anti-apoptosis in the immune system and NF-kB signaling to promote neurite outgrowth; and FAIM long (FAIM-L), which is expressed only in neurons and exerts an anti-apoptotic affect [49]. The instruments we used to estimate the causal effect of *FAIM* on glioma risk (rs361073 in the amygdala and rs361074 in the nucleus accumbens, LD r^2^ = 0.99) were associated with an exon skipping event that is present in two transcripts, ENST00000338446 and ENST00000470889. Furthermore, the CancerSplicingQTL project has identified this variant as a cancer risk sQTL affecting exon 3 (chr3 position 138 329 446 in GRCh38) for, among others, GB, low-grade glioma, breast cancer, lung adenocarcinoma and squamous cell carcinoma [50]. This exon is present in the two previously stated transcripts, whereby the latter transcript, ENST00000470889, contains a retained intron but otherwise contains the same configuration of exons as ENST00000338446. Transcript ENST00000338446 is a protein-coding transcript and results in a relatively novel and understudied protein named by Coccia, *et al* as FAIM-S_2a because the protein contains the same sequences as FAIM-S with an extra exon [51]. Similarly, Coccia, *et al* found that FAIM-S_2a is ubiquitously expressed and increases neurite outgrowth, but also found that the protein can localise to the nucleus with potentially additional functions. This FAIM isoform and the splice variant, which is associated with it, have thus been implicated in cancer risk; however, our results highlight that this variant may instead be putatively causal for, and not just associated with, glioma risk. More research into this splice isoform is required to ascertain its function and whether it is a risk event for gliomagenesis.

*HBEGF* is a member of the EGF family of growth factors which is involved in the MAPK, STAT and PI3K/AKT pathways [52, 53]. These molecular pathways have roles in regulating biological processes, such as positive regulation of cell growth, migration, and proliferation [54]. The role of *HBEGF* in the CNS is well characterised: it is highly expressed throughout; has roles in both the developing and adult brain, with studies indicating *HBEGF* contributes to glial and stem cell proliferation and is a ligand for EGFR [55, 56]. Furthermore, expression of *HBEGF* has been shown to be increased significantly in many human cancer types, and *HBEGF* mRNA expression has been shown to be increased two- to 5-fold in GB cell lines compared to control brain tissue [57]. We found no significant differences between control and any glioma subtypes but did find differential expression between GB and non-GB subtypes in two independent datasets (Supplementary Fig. S4A and B) [24, 25]. We also found that the instrumented *HBEGF* eQTL (rs4150197) had no associations with cancer risk or any putative risk factors for glioma (Supplementary Table S14). We observed no evidence for an effect through non-canonical transcripts or proteins. Altogether, our causal evidence implicates the expression of *HBEGF* in the brain as a potentially novel biomarker, though more research would be required to ascertain its role. Given that there are drugs which target *HBEGF*, glioma and *HBEGF* may make for an interesting target-indication pair for future studies.

*SLC8A1* is a gene whose protein product (sometimes known as sodium/calcium exchanger 1, NCX1) mediates the exchange of calcium and sodium across the cell membrane. The gene is ubiquitously expressed, and apart from its role in calcium homeostasis, it also plays a key role in neuronal function [58]. The instrument we used to proxy *SLC8A1* splice variant expression associated with a splice event at junction seven (chr2: 40170349-40174825) linking exons 10 and 12 and, according to GTEx, this event was measured mostly within the brain tissues (with non-zero read counts also in the heart, colon, female reproductive tissues, oesophagus, kidney, and bladder). Tissue and cell type-specific isoform expression of *SLC8A1* has been observed, with specific SLCA81 isoforms mediating calcium homeostasis in neurons and astrocytes [59]. A mounting body of evidence has pointed to Ca^2+^ signalling as driving gliomagenesis by way of altering cell competition mechanisms that potentially aid motility and invasion of GB cells [60, 61]. Our MR results show that increased *SLC8A1* isoform mRNA expression is putatively protective against glioma; however, a previous study found that the cytotoxic effect of amiloride, a drug which kills glioma cells, occurs due to dual inhibition of sodium-proton exchanger (NHE1) and the NCX1.1 isoform, leading to glioma cell demise through increased Ca^2+^ concentration [62]. Although our findings implicate *SLC8A1* in gliomagenesis and as a putative therapeutic target, further research is required to ascertain which isoform is potentially driving gliomagenesis, and through which mechanisms this occurs.

*RTEL1* is a gene which encodes for a DNA helicase responsible for the elongation of telomeres, and is known to interact with the telosome complex, a group of proteins which protect the telomere caps of DNA [63]. *RTEL1* has previously been identified as a risk factor for glioma, and studies have tried to identify whether telomere length has an established causative effect on the risk of glioma, although no definitive conclusion has been reached [9, 11, 18–20]. Wang *et al*. found that relative telomere length and *RTEL1* mRNA correlated with predicted worse progression in glioma patients, but not in meningioma patients (*P* < 0.01), and that increased relative telomere length was weakly correlated with *RTEL1* mRNA expression (R^2^ = 0.248, *P* < 0.001) [64]. *RTEL1*-associated sQTLs were associated with an exon-skipping event (GTEx intron ID: 63689132:63689750:clu_27064) present only in *RTEL1* transcript ENST00000425905 (*P* = 4.23 × 10^−75^ to 6.64 × 10^−18^). Therefore, it may be the case that previous conflicting evidence on the directionality of the effect of *RTEL1* expression on glioma risk may be confounded due to non-canonical transcript variants; though, further studies would be required to ascertain if this is the case. Furthermore, we observed that *RTEL1* sQTLs rs6063202 and rs2297440, were found to be strongly associated to GB (*P* = 1.00 × 10^−13^ and 4.00 × 10^−46^, respectively) and with allergy-related traits (*P* = 4.78 × 10^−8^ to 7.84 × 10^−19^, Supplementary Table S12). These results beget further study and could help to understand the links between allergies, glioma risk and *RTEL1.*

Variants can act via multiple molecular QTL pathways and are not mutually exclusive; variants affecting gene expression can also be associated with alternative gene splicing of the same genes. As eQTL and sQTL are both measured by quantifying mRNA levels, this was not unexpected [65]. Using colocalisation, we found that the effect of *HEATR3* appears to be eQTL-driven (*P* = 1.95 × 10^−10^ to 6.74 × 10^−12^) despite finding a robust causal effect in the alternative splicing analysis (*P* = 1.29 × 10^−10^) (Supplementary Table S19). The causal effect is likely driven through expression of the canonical gene isoform, which is incorrectly measured as an alternative splice isoform due to the way in which eQTL and sQTL are measured [66]. Furthermore, some probes for mRNA will detect commonly splice variants as canonical transcripts, which are included in ‘bulk tissue’ eQTL analysis. This can lead to the false assumption that both expression and splicing events are driving the causal effect [67]. We also found that the causal effect of *EGFR* expression in the cerebellum (*P* = 1.60 × 10^−7^ to 2.89 × 10^−31^) showed evidence of colocalisation with the casual effect of EGFR protein abundance in the cortex (*P* = 3.82 × 10^−7^ to 2.19 × 10^−29^) (H_4_ = 99%). Causal *cis-*pQTLs have previously been found to colocalise with causal *cis-*eQTLs, which implicates that variants may have a role in the variation of both molecular traits [68].

The expression and splice isoform QTLs used in this study were derived from multiple different brain regions, and, as such, may provide some insight into aetiologically important brain regions for glioma risk. However, we would like to provide some caution in interpreting our results in this context. Specifically, it has been previously observed that eQTLs which associate with gene expression in different brain regions are likely to be both cis-acting and have the same effect on the expression of that gene in those regions [69]; therefore, the effect of that gene on glioma aetiology may not be region specific, but instead act more broadly within the brain or even the periphery. Further analyses would be required to provide further evidence for such a brain region-specific link using, for example, RNA-Seq data [70].

Finally, the QTLs used in this study were derived from bulk tissue composed of many cell types. Whilst it would have been advantageous to perform single-cell eQTL (sc-eQTL) MR analysis to identify cell types that may be implicated in the risk of glioma, this would not be plausible with the sc-eQTL datasets currently available, due to very small sample sizes. Additionally, previous work by Byrois *et al*. showed that the majority (77%–100%) of SNP-gene pairs from sc-eQTL replicate in bulk cortical eQTLs [71].

### Strengths

We aimed to identify molecular traits with the most robust evidence for causal roles in glioma risk, by combining strong MR evidence with colocalisation and Steiger filtering. Assessing different ‘omics’ data sources can be beneficial to understand the development of complex traits and for drug target-related analyses; each QTL provides an insight into different molecular processes [13, 14].

In addition to the ‘combined’ glioma GWAS, we used GB-only and non-GB-only caseloads in order to reduce the heterogeneity of our results and determine if our robust causal results are shared across all glioma or limited to one subtype.

To test if the causal effects were driven via putative glioma risk factors, we tested for potential pleiotropic effects in known risk factors for glioma: allergies, asthma, altered LDLc levels and alcohol consumption. This may provide evidence of horizontal pleiotropy, which may make it harder to draw decisive conclusions of causality [72, 73] However, this would require follow up studies to investigate which could lead to potentially elucidating how these putative risk factors and molecular traits are linked to glioma.

### Limitations

Despite using relatively large datasets, our analyses are still likely to suffer from limited statistical power due to restricted sample sizes, particularly in the GTEx dataset (n = 114 to 209). This might lead to some important causal effects not being identified. Additionally, as we used the GTEx sGenes, only the top splice event per gene was measured, and therefore there were many splice events which were not tested in our MR analysis.

Most of the MR analyses used a single variant instrument, which restricts the type of sensitivity analyses that could be performed; however, this is a common phenomenon observed when conducting MR with molecular traits [73].

Our analysis is limited to individuals with European ancestry; therefore, it will be important to extend these analyses to individuals of alternative ancestries as such data becomes available [74]. Previous GWAS and observational studies in non-European cohorts have found novel associations which we did not find in our study e.g. *GSTP1*, which may illustrate that the loci appear to be population specific, or may be observational, and not causal [75, 76].

## Conclusion

We conducted a robust multi-omic causal analysis for gene and protein molecular traits in brain tissue on glioma risk. We used brain region-specific molecular data and glioma subtype information to explore the nature of the causal relationships identified. Here, we combined MR with colocalisation and Steiger filtering to increase the robustness of our results.

We provide robust multi-omic causal evidence for 13 previously implicated genes (or their products) which affect the risk of glioma. We also present novel evidence for a causal effect of increased *HBEGF* expression in cortex on increased risk of all glioma, novel evidence for causal effects of *CEP192*, *FAIM* and *SLC8A1* splicing in multiple tissues on variable risk of all glioma and GB and novel evidence for a causal effect of increased protein abundance of D2HGDH in cortex on decreased risk of all glioma in the cortex.

We focused on the novel causal effects and used multiple investigative methods to provide evidence to understand the biological mechanisms that these genes and proteins may play in the risk of glioma.

The results of this study provide insight to the molecular pathways implicated in the process of gliomagenesis, which may be utilised in tangible translational opportunities for potential drug repurposing or novel drug discovery in the prevention of gliomagenesis.

## Material and methods

**Figure 4:**
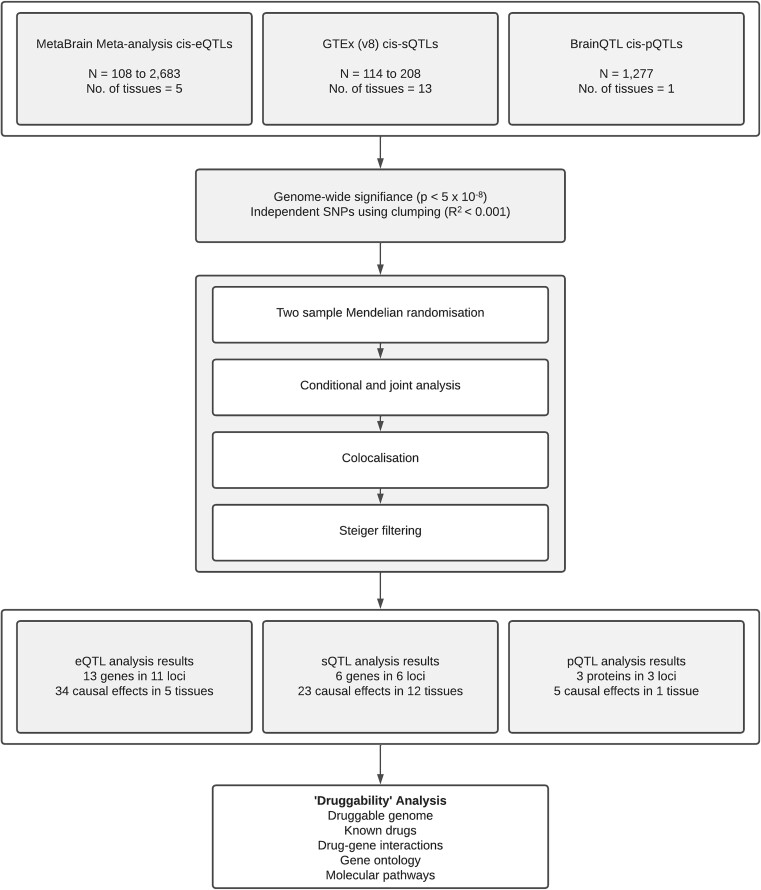
Flowchart showing the pipeline used in the multi-omic analysis.

A diagram of the workflow described in this section can be found in Fig. 4.

### Study datasets

We used QTL summary statistics from GWAS of gene expression, protein abundance and alternative splicing levels in various brain regions described below. We performed a hypothesis-free analysis, including all genes, splicing isoforms, or proteins which had valid QTLs that passed our selection criteria (see below).

Genetic data associated with gene expression were obtained from MetaBrain, a meta-analysis of 14 eQTL datasets derived from 3659 samples from 2683 individuals of European ancestry in five CNS regions: the basal ganglia (n = 208), cerebellum (n = 492), cortex (n = 2683), hippocampus (n = 168), and spinal cord (n = 108) (Supplementary Table S20) [16].

The sQTL data were retrieved from GTEx Portal (v8 release). We used sGenes provided by GTEx, generated using their quality control (QC) protocol from a European-American population. sQTL were extracted from 13 regions of the CNS: amygdala (n = 129), anterior cingulate cortex (BA24) (n = 147), caudate (n = 194), cerebellar hemisphere (n = 175), cerebellum (n = 209), cortex (n = 205), frontal cortex (BA9) (n = 175), hippocampus (n = 165), hypothalamus (n = 170), nucleus accumbens (n = 202), putamen (n = 170), spinal cord (n = 126), and substantia nigra (n = 114) (Supplementary Table S21) [17].

Finally, we included pQTL data retrieved from BrainQTL, derived from 1277 European ancestry individuals from the religious orders study and memory and ageing projects (ROSMAP), The Arizona study of aging and neurodegenerative disorders, The Baltimore longitudinal study of aging and Mount Sinai and JJ Peters VA Medical Centre Brain Bank [77–81]. pQTL were extracted from six regions of the cerebral cortex: dorsolateral prefrontal cortex (n = 793), premotor cortex (n = 123), middle frontal gyrus (n = 46), temporal cortex (n = 123), precuneus (n = 46) and parahippocampal gyrus (n = 146) (Supplementary Table S22). All regions were meta-analysed as one sample [82].

Glioma summary-level data were derived from a meta-analysis of eight constituent glioma GWAS consisted of 6191 GB cases, 5819 non-GB cases, 12 496 combined cases and 18 190 controls [6]. We therefore used three outcomes throughout our analyses: the GB-only case load, the non-GB-only case load, and the combined case load (defined as ‘all glioma’). Although glioma is highly heterogeneous, we did this to increase our statistical power by using larger sample sizes. The non-GB cases analysed in this meta-analysis were not explicitly classified but consists mainly of astrocytoma and oligodendroglioma diagnosed patients.

This study uses previously published summary-level data and thus contains no patient identifiable data. Ethical approval and informed consent from each participant were given and can be found where the dataset was initially described. All procedures performed in studies involving human participants were done in accordance with the ethical standards of the institutional or national research committee and with 1964 Helsinki declaration.

### Instrument selection

We identified *cis-*acting (within 1 Mb of the gene coding region) QTLs which met genome-wide significance (*P* < 5 × 10^−8^). *Trans-*acting QTLs were excluded from the analysis because of the increased likelihood of horizontal pleiotropy, due to their distant location from the gene whose variation they alter. Instruments were selected to be in linkage disequilibrium (R^2^ < 0.001) to ensure independence. The complete list of variants used to construct IVs can be found in Supplementary Table S23.

### Two sample Mendelian randomisation

We used a two-sample MR framework to estimate the causal effect of genetically proxied gene expression, protein abundance and alternative splicing on genetic liability to glioma risk. Two sample MR was performed using the TwoSampleMR package from MRBase [83]. MR estimates were generated using the Wald ratio method for instruments consisting of single variants and inverse variance weighted (IVW) method for instruments comprising of multiple variants [84]. MR estimates were transformed and presented throughout as odds ratios (OR) and were scaled to reflect one standard deviation increase in the respective molecular trait. Following MR analysis, the results had to meet a Bonferroni-corrected *P*-value threshold (0.05/number of tests performed) to adjust for multiple testing.

MR has three assumptions which must hold to produce an unbiased estimate. Firstly, the genetic instrument must associate with the exposure (‘relevance’). We tested this by generating the F-statistic for each instrument, where an F-statistic > 10 is evidence against weak instrument bias and filtering out instruments which did not surpass this threshold [72, 85]. The second assumption is that the genetic instrument does not share a common cause with the outcome (‘independence’). In QTL analyses, it is uncommon to test for this assumption using methods, such as MR-Egger, due to the general occurrence of single variant instruments. However, we attempted to rule out that linkage disequilibrium can explain the shared effects on molecular trait and outcome (one aspect of this assumption) by using genetic colocalisation, which determines whether the molecular trait and glioma share the same causal variant, a necessary (though not sufficient) condition for causality. Using colocalisation in this way has been posited to at least eliminate some unreliable associations when standard follow up sensitivity analyses to evaluate the presence of horizontal pleiotropy (such as MR-Egger) are unavailable [72, 73].

**Figure 5:**
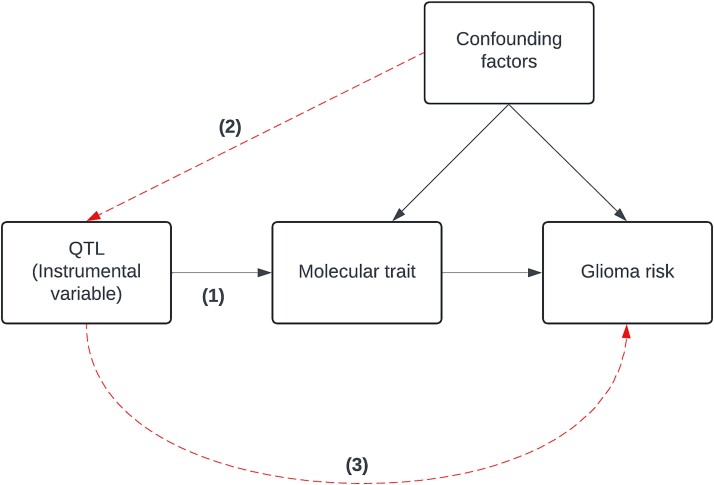
Directed acyclic graph (DAG) showing a visual representation of potential causal relationships. This DAG also shows the three assumptions of Mendelian randomisation. (1) The genetic variant(s) being used as an instrument is associated robustly with the exposure. (2) The instrument is independent of measured and unmeasured confounding factors of the association between exposure and outcome. (3) There must be no independent pathway between the instrument and outcome other than through the exposure.

The final assumption is that the genetic instrument affects the outcome only through the effect of the exposure of interest (‘exclusion restriction’). This assumption, also known as horizontal pleiotropy, is difficult to assess with single variant instruments, as is common for ‘omic’ variables. This was tested by investigating whether there was causal evidence linking the molecular trait of interest with putative risk factors for glioma. The presence of an association between the molecular trait and glioma risk factors may then be evidence of potential horizontal pleiotropy (Fig. 5) [72].

For brevity, we refer to our MR results as causal genes (or causal proteins); however, we recognise that further studies and evidence will be required to validate our results.

### Colocalisation

We tested for colocalisation between the loci of each molecular trait and glioma subtype which had an MR result that passed the Bonferroni-corrected *P*-value threshold, using a reference panel derived from a subset of 10 000 unrelated individuals of European ancestry from the UK10K project [86]. We extracted *cis-*regions consisting of all variants within a 1 Mb window around the gene coding regions [87]. We performed genetic colocalisation using PWCoCo, a method which combines GCTA-COJO and colocalisation (coloc R package) to ensure the single causal variant assumption of colocalisation holds [87–89]. Briefly, the colocalisation analysis will provide evidence for five possible hypotheses of the potential existence of a shared causal variant between the QTL *cis*-region and the corresponding region in the glioma GWAS. We defined evidence of colocalisation when the fourth hypothesis (H_4_)—i.e. the hypothesis where a shared causal variant does exist—is greater than 80%. For further details, please see Giambartolomei, *et al*. [87].

### Steiger filtering

For causal effects which had strong evidence of colocalisation, we applied Steiger filtering to test if the instrument explains more of the variance in the outcome than the exposure [73]. If this is true, then it is unlikely that such an instrument is valid under the assumptions required by MR. Results of Steiger filtering are presented as a one of three categorical variables; ‘pass’ if Steiger *P* < 0.05 and the instrument explains more of the variance in the exposure than the outcome, ‘fail’ if Steiger *P* < 0.05 and the instrument explains more of the variance in the outcome than the exposure, and ‘uncertain’ if *P* ≥ 0.05. As Steiger filtering is sensitive to sample size, we still consider those results where the proportion of variance explained is greater in the exposure than the outcome with an ‘uncertain’ *P* value.

### Evidence thresholds for multi-omic analyses

Our main results comprised of those which passed the Bonferroni correction *P*-value, had strong colocalisation evidence (H_4_ ≥ 80%) and passed Steiger filtering sensitivity analysis, as they have the most robust evidence. Since Steiger filtering is sensitive to sample sizes, the analysis may be liable to false negatives if one dataset is better powered than the other; therefore, given the relatively small samples in the QTL datasets, we included results which had ‘uncertain’ evidence but with the caveat that these results may be less robust.

### Overlap in signals between molecular traits

To determine whether there was any potential overlap in signals between molecular traits, we looked at all MR results for our molecular traits with robust evidence in all brain regions, regardless of *P*-value. We then filtered these results using a more lenient Bonferroni-corrected *P*-value (*P* < 4.35 × 10^−4^) to determine whether any were potentially overlapping signals which failed to pass the primary analysis due to insufficient statistical power.

### Specificity analysis

To annotate the potential causal pathway between the molecular trait and glioma, we used two different approaches: Performing MR analyses between the molecular traits and previously reported risk factors for glioma, and a hypothesis-free PheWAS screen. To determine whether the robust causal effects were driven through putative glioma risk factors, we investigated all risk factors with genetic liability (allergies (ICD10: Z91.0—Personal history of allergy, other than to drugs and biological substances, and, ICD10: Z88.8—Personal history of allergy to other drugs, medicaments and biological substances), asthma (ICD10: J45.9—Asthma, unspecified), and continuous traits (LDLc levels and alcohol consumption). These risk factors were selected based on potential evidence linking that risk factor to glioma in the literature, if there was GWAS data available for that risk factor in the IEU OpenGWAS project database [90]. To perform the hypothesis-free search for potential causal pathways, we used the IEU OpenGWAS project PheWAS tool and PhenoScanner V2 to search for phenotypes which were strongly associated (*P* < 5 × 10^−8^) with lead variants used to instrument the robust causal effects [21, 22, 91]. Differential expression in molecular traits

We explored whether there was differential expression in the genes for which there was robust evidence that expression had a causal effect on at least one glioma subtype, between subtypes of glioma and control patients using the GlioVis resource [23].

We investigated the differences in the expression of splicing variants in brain regions in the genes for which there was evidence that gene-splicing had a causal effect on at least one glioma subtype, using the GTEx portal [17].

### Single cell-specific expression patterns

As we used bulk brain tissue for the main analysis, we sought to identify gene expression levels in specific cell types using single-cell data from the HPA (v23) [26]. We excluded broadly expressed genes and genes whose expression were not detected in the brain.

### Annotation of drug tractability for robustly causal genes and proteins

We annotated robust results which passed sensitivity analyses with evidence of drug tractability i.e. how likely a gene and its gene product are to be a valid drug target, to build evidence for the prioritisation of genes and proteins as candidate targets. We searched for our robust causal genes and proteins in the Open Targets platform, a large-scale database that uses genetic and genomic data for systematic drug target identification and prioritisation, to search for gene ontology terms, pathways, and gene interactions [27, 28] We also used DGIdb to search for drug-gene interactions and genome ‘druggability’ [29].

## Supplementary Material

Manhattan_Plot_eQTL_(Figure_S1)_ddae168

Manhattan_Plot_sQTL_(Figure_S2)_ddae168

Manhattan_Plot_pQTL_(Figure_S3)_ddae168

Rembrandt_HBEGF_Differential_Expression_(Figure_S4a)_ddae168

Rembrandt_D2HGDH_Differential_Expression_(Figure_S4b)_ddae168

TCGA_HBEGF_Differential_Expression_(Figure_S4c)_ddae168

TCGA_D2HGDH_Differential_Expression_(Figure_S4d)_ddae168

Supplementary_Tables_ddae168

STROBE_MR_Checklist_ddae168

## Data Availability

The MetaBrain meta-analyses eQTL data can be accessed at https://www.metabrain.nl/. The GTEx sQTL v8 data can be accessed at https://gtexportal.org/home/datasets. The BrainQTL pQTL data can be found at https://www.synapse.org/Synapse:syn51154733. The glioma data may be accessed under the European Genome-phenome Archive accession number EGAD00010001657 (https://www.ebi.ac.uk/ega/datasets/EGAD00010001657).
