# Supplementary figures and images for: Brain multi-omic Mendelian randomisation to identify novel drug targets for gliomagenesis
Source: Hum Mol Genet. 2024 Nov 20;34(2):178–92. doi: 10.1093/hmg/ddae168 (PMC11780873; doi:10.1093/hmg/ddae168)

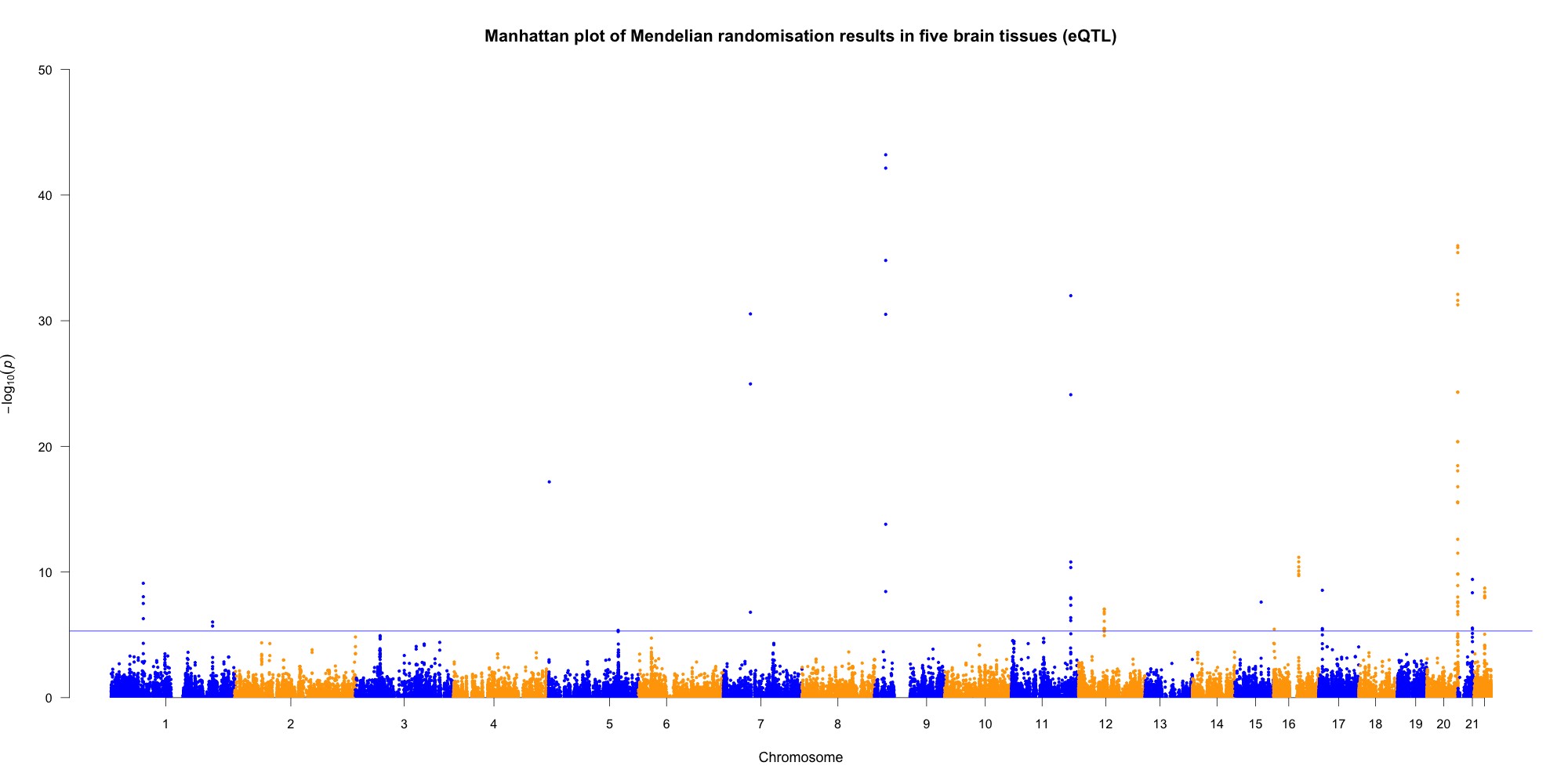

Supplement: Manhattan_Plot_eQTL_(Figure_S1)_ddae168 [file manhattan_plot_eqtl_(figure_s1)_ddae168.jpeg]

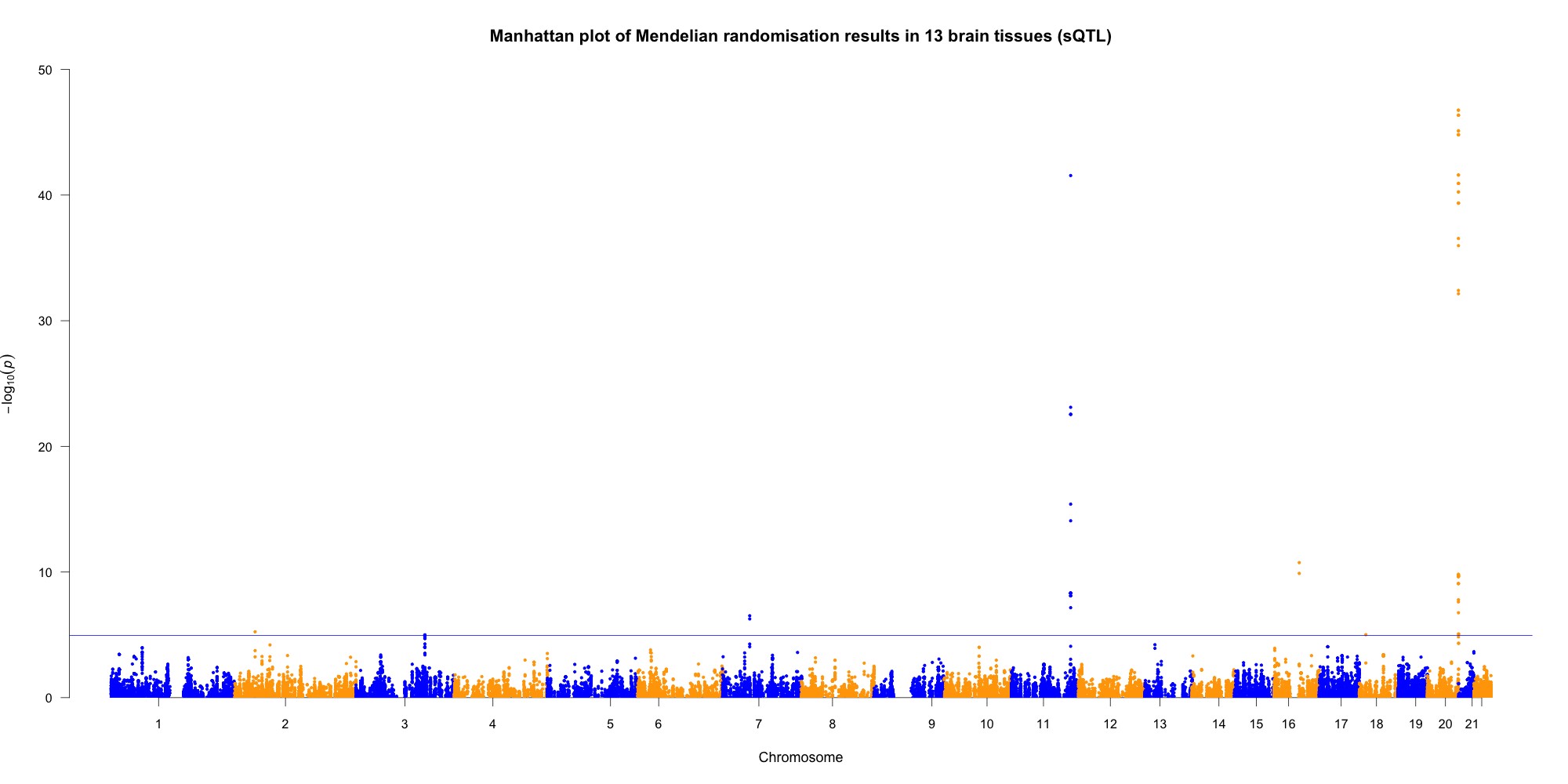

Supplement: Manhattan_Plot_sQTL_(Figure_S2)_ddae168 [file manhattan_plot_sqtl_(figure_s2)_ddae168.jpeg]

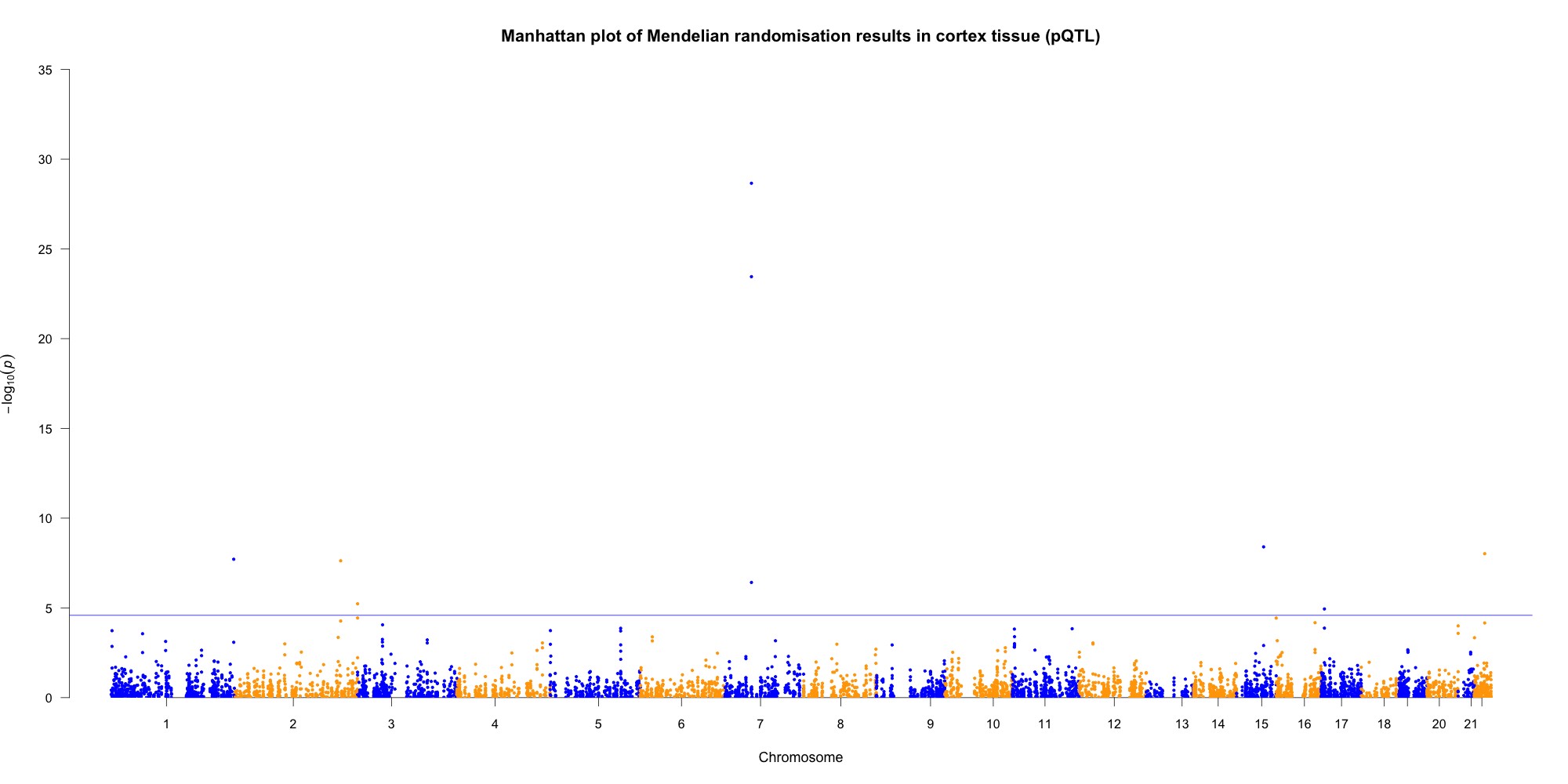

Supplement: Manhattan_Plot_pQTL_(Figure_S3)_ddae168 [file manhattan_plot_pqtl_(figure_s3)_ddae168.jpeg]

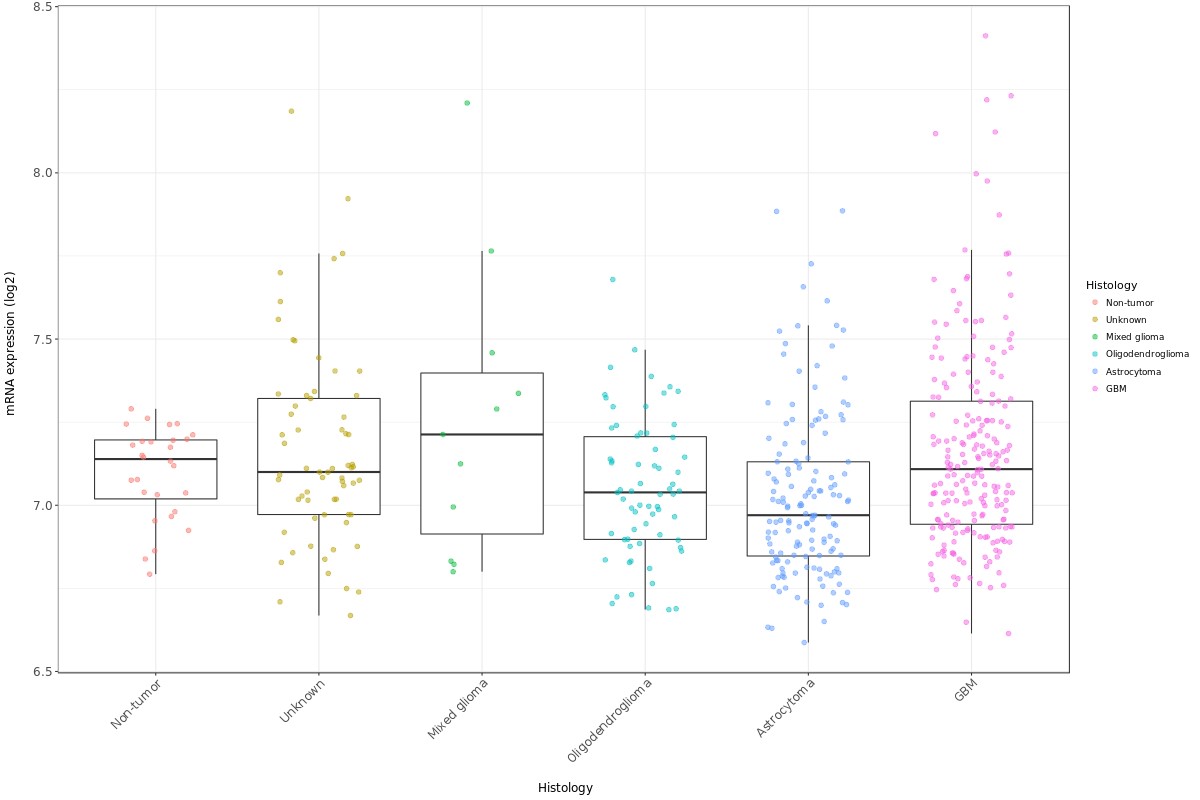

Supplement: Rembrandt_HBEGF_Differential_Expression_(Figure_S4a)_ddae168 [file rembrandt_hbegf_differential_expression_(figure_s4a)_ddae168.jpeg]

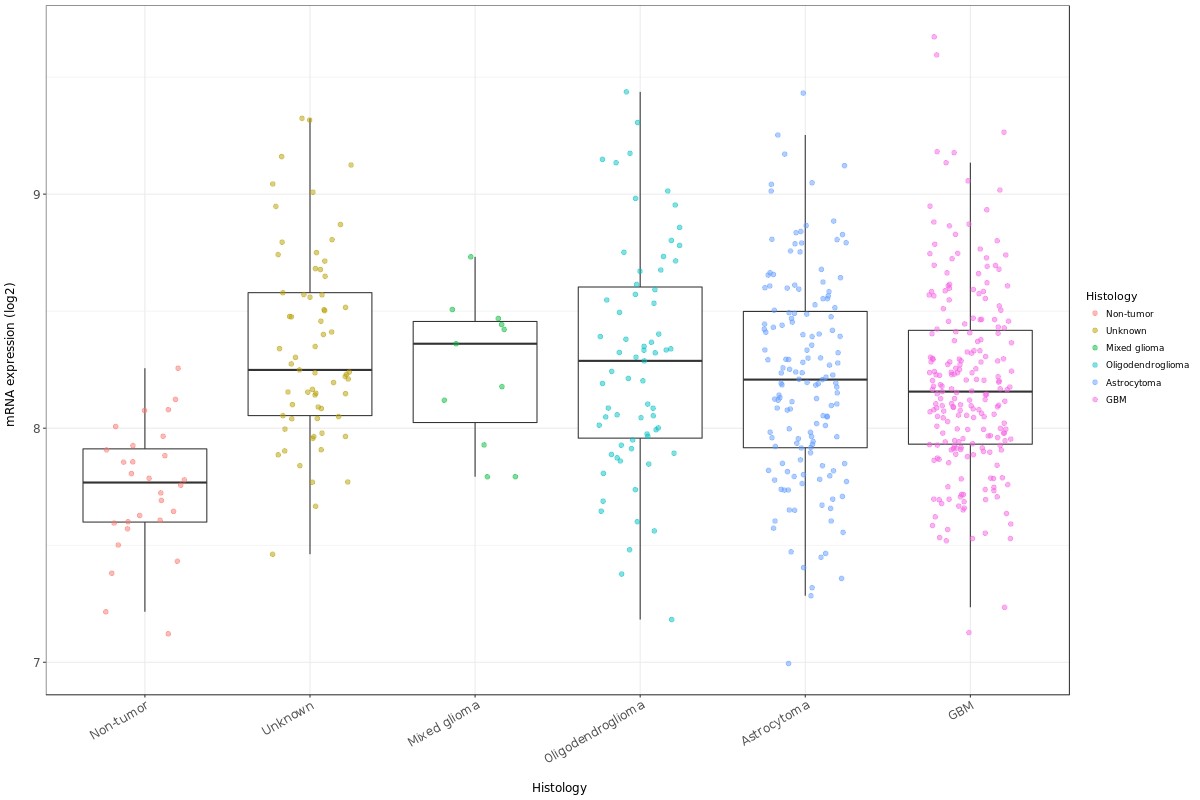

Supplement: Rembrandt_D2HGDH_Differential_Expression_(Figure_S4b)_ddae168 [file rembrandt_d2hgdh_differential_expression_(figure_s4b)_ddae168.jpeg]

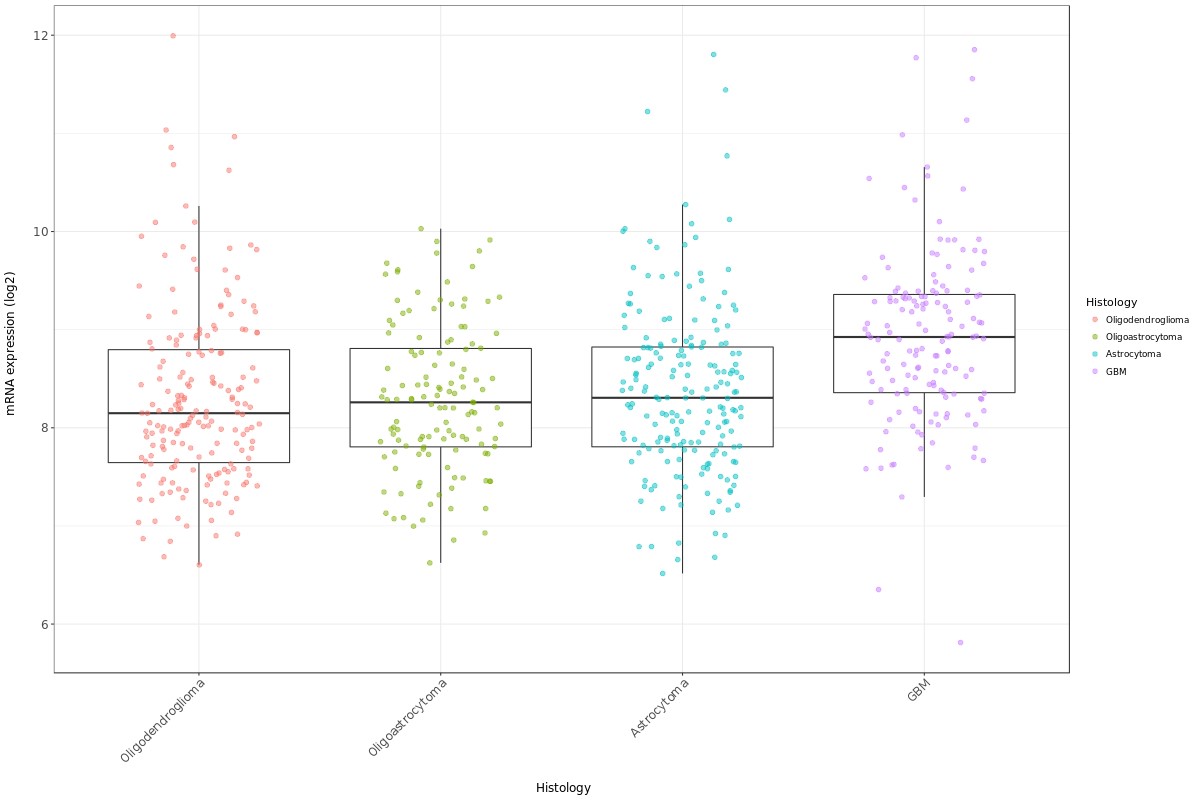

Supplement: TCGA_HBEGF_Differential_Expression_(Figure_S4c)_ddae168 [file tcga_hbegf_differential_expression_(figure_s4c)_ddae168.jpeg]

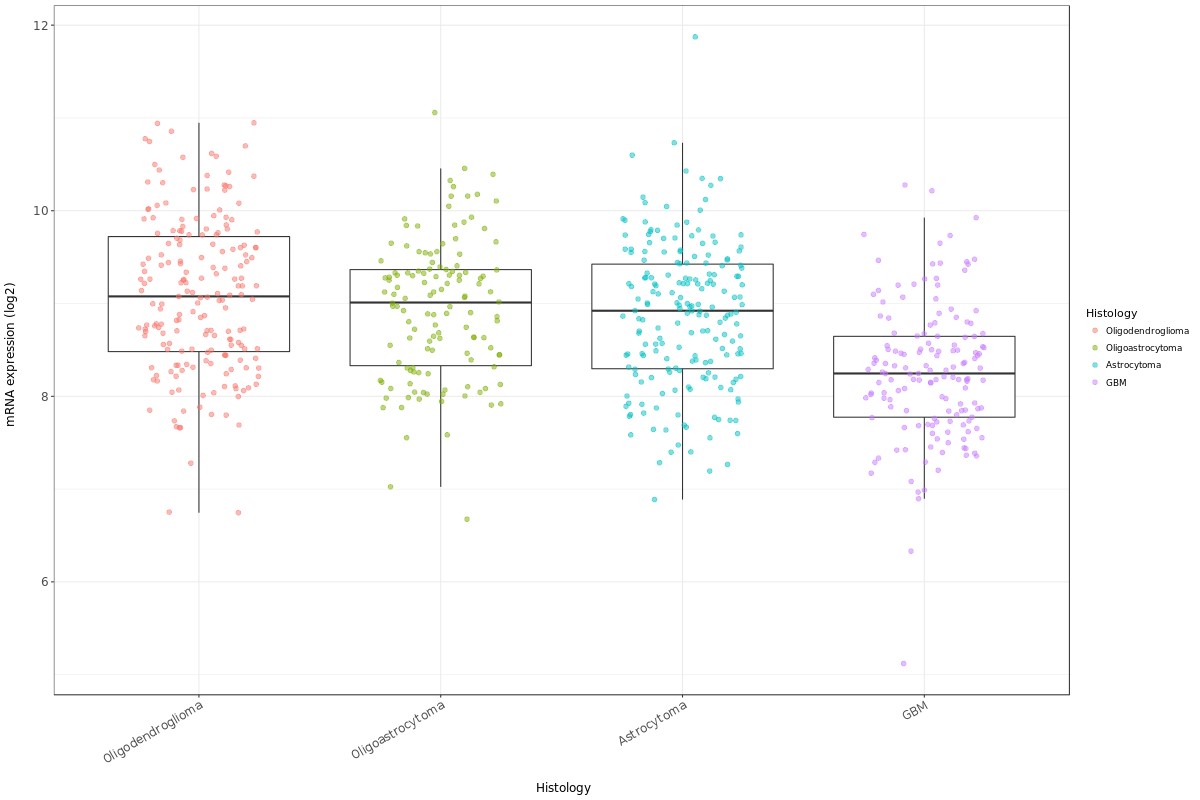

Supplement: TCGA_D2HGDH_Differential_Expression_(Figure_S4d)_ddae168 [file tcga_d2hgdh_differential_expression_(figure_s4d)_ddae168.jpeg]
