# Supplementary material for: Brain multi-omic Mendelian randomisation to identify novel drug targets for gliomagenesis
Source: Hum Mol Genet. 2024 Nov 20;34(2):178–92. doi: 10.1093/hmg/ddae168 (PMC11780873; doi:10.1093/hmg/ddae168)
Supplement: STROBE_MR_Checklist_ddae168 [file strobe_mr_checklist_ddae168.docx]

**STROBE-MR checklist of recommended items to address in reports of Mendelian randomization studies**

| **Item No.** | **Section** | **Checklist item** | **Page No.** | **Relevant text from manuscript** |
| --- | --- | --- | --- | --- |
| 1 | **TITLE and ABSTRACT** | Indicate Mendelian randomization (MR) as the study’s design in the title and/or the abstract if that is a main purpose of the study | 1,2 | Multi-omics Mendelian randomisation using expression, splicing and protein quantitative trait loci: identification of novel drug targets for gliomagenesis. We performed two-sample Mendelian randomisation and genetic colocalisation of a large panel of molecular traits on glioma. |
|  | **INTRODUCTION** |  |  |  |
| 2 | **Background** | Explain the scientific background and rationale for the reported study. What is the exposure? Is a potential causal relationship between exposure and outcome plausible? Justify why MR is a helpful method to address the study question | 3 | Such genetic variants associated with these molecular traits are known as quantitative trait loci (QTL), and examples of these include measuring relative gene expression levels (eQTLs), splicing variation (sQTLs) and protein abundance (pQTLs). |
| 3 | **Objectives** | State specific objectives clearly, including pre-specified causal hypotheses (if any). State that MR is a method that, under specific assumptions, intends to estimate causal effects | 3 | …with the aim to identify causal evidence for aetiologically important genes for gliomagenesis. |
|  | **METHODS** |  |  |  |
| 4 | **Study design and data sources** | Present key elements of the study design early in the article. Consider including a table listing sources of data for all phases of the study. For each data source contributing to the analysis, describe the following: |  |  |
|  | a) | Setting: Describe the study design and the underlying population, if possible. Describe the setting, locations, and relevant dates, including periods of recruitment, exposure, follow-up, and data collection, when available. | 5 | Gene expression data were taken from MetaBrain, a meta-analysis of 14 eQTL datasets derived from 3,659 samples from 2,683 individuals of European ancestry in five CNS tissues: the basal ganglia (n=208), cerebellum (n=492), cortex (n=2,683), hippocampus (n=168), and spinal cord (n=108) (Supplementary Table S1). The sQTL data were taken from GTEx Portal (v8 release). We used sGenes provided by GTEx, generated using their QC protocol, from a primarily European-American (85.3%) population. sQTL were extracted from 13 regions of the CNS: amygdala (n=129), anterior cingulate cortex (BA24) (n=147), caudate (n=194), cerebellar hemisphere (n=175), cerebellum (n=209), cortex (n=205), frontal cortex (BA9) (n=175), hippocampus (n=165), hypothalamus (n=170), nucleus accumbens (n=202), putamen (n=170), spinal cord (n=126), and substantia nigra (n=114) (Supplementary Table S1). Finally, we included pQTL data retrieved from BrainQTL, derived from European ancestry individuals in the religious orders study and memory and ageing projects. pQTL were extracted from the dorsolateral pre-frontal cortex of 330 individuals (Supplementary Table S1). |
|  | b) | Participants: Give the eligibility criteria, and the sources and methods of selection of participants. Report the sample size, and whether any power or sample size calculations were carried out prior to the main analysis | - |  |
|  | c) | Describe measurement, quality control and selection of genetic variants | 6 | We identified cis-acting (within 1Mb of the gene coding region) QTLs which met genome-wide significance (P < 5x10-8). Trans-acting QTLs were excluded from the analysis because of the increased likelihood of horizontal pleiotropy, due to their distant location from the gene whose variation they alter. Instruments were selected to be in linkage disequilibrium (R2 < 0.001) to ensure independence. |
|  | d) | For each exposure, outcome, and other relevant variables, describe methods of assessment and diagnostic criteria for diseases | 5 | Glioma summary-level data were derived from a meta-analysis of eight constituent glioma GWAS consisted of 6,191 glioblastoma (GB) cases, 5,819 non-GB cases, 12,496 combined cases and 18,190 controls. We therefore used three outcomes throughout our analyses: the GB-only case load, the non-GB-only case load, and the combined case load (defined as ‘all glioma’). |
|  | e) | Provide details of ethics committee approval and participant informed consent, if relevant | 6 | Ethical approval and informed consent from each participant were given and can be found where the dataset was initially described. All procedures performed in studies involving human participants were done in accordance with the ethical standards of the institutional or national research committee and with 1964 Helsinki declaration |
| 5 | **Assumptions** | Explicitly state the three core IV assumptions for the main analysis (relevance, independence and exclusion restriction) as well assumptions for any additional or sensitivity analysis | 6,7 | MR has three assumptions which must hold to produce an unbiased estimate… |
| 6 | **Statistical methods: main analysis** | Describe statistical methods and statistics used |  |  |
|  | a) | Describe how quantitative variables were handled in the analyses (i.e., scale, units, model) | 6 | MR estimates were transformed and presented throughout as odds ratios (OR) and were scaled to reflect one standard deviation increase in the respective molecular trait. |
|  | b) | Describe how genetic variants were handled in the analyses and, if applicable, how their weights were selected | - |  |
|  | c) | Describe the MR estimator (e.g. two-stage least squares, Wald ratio) and related statistics. Detail the included covariates and, in case of two-sample MR, whether the same covariate set was used for adjustment in the two samples | 6 | MR estimates were generated using the Wald ratio method for instruments consisting of single SNPs and inverse variance weighted (IVW) method for instruments comprising of multiple SNPs. |
|  | d) | Explain how missing data were addressed | - |  |
|  | e) | If applicable, indicate how multiple testing was addressed | 6 | Following MR analysis, the results had to meet a Bonferroni-corrected P value threshold (0.05/number of tests performed) to adjust for multiple testing. |
| 7 | **Assessment of assumptions** | Describe any methods or prior knowledge used to assess the assumptions or justify their validity | 6,7 |  |
| 8 | **Sensitivity analyses and additional analyses** | Describe any sensitivity analyses or additional analyses performed (e.g. comparison of effect estimates from different approaches, independent replication, bias analytic techniques, validation of instruments, simulations) | 7 | Using colocalisation in this way has been posited to at least eliminate some unreliable associations when standard follow up sensitivity analyses to evaluate the presence of horizontal pleiotropy (such as MR-Egger) are unavailable. |
| 9 | **Software and pre-registration** |  |  |  |
|  | a) | Name statistical software and package(s), including version and settings used | 10 |  |
|  | b) | State whether the study protocol and details were pre-registered (as well as when and where) | - |  |
|  | **RESULTS** |  |  |  |
| 10 | **Descriptive data** |  |  |  |
|  | a) | Report the numbers of individuals at each stage of included studies and reasons for exclusion. Consider use of a flow diagram | - |  |
|  | b) | Report summary statistics for phenotypic exposure(s), outcome(s), and other relevant variables (e.g. means, SDs, proportions) | - |  |
|  | c) | If the data sources include meta-analyses of previous studies, provide the assessments of heterogeneity across these studies | - |  |
|  | d) | For two-sample MR:  i.  Provide justification of the similarity of the genetic variant-exposure associations between the exposure and outcome samples  ii.  Provide information on the number of individuals who overlap between the exposure and outcome studies | - |  |
| 11 | **Main results** |  |  |  |
|  | a) | Report the associations between genetic variant and exposure, and between genetic variant and outcome, preferably on an interpretable scale |  | Supplementary Table 2 |
|  | b) | Report MR estimates of the relationship between exposure and outcome, and the measures of uncertainty from the MR analysis, on an interpretable scale, such as odds ratio or relative risk per SD difference |  | Table 1,2 |
|  | c) | If relevant, consider translating estimates of relative risk into absolute risk for a meaningful time period | - |  |
|  | d) | Consider plots to visualize results (e.g. forest plot, scatterplot of associations between genetic variants and outcome versus between genetic variants and exposure) |  | Figure 3,4 |
| 12 | **Assessment of assumptions** |  |  |  |
|  | a) | Report the assessment of the validity of the assumptions | 6,7 | We tested this by generating the F-statistic for each instrument, where an F-statistic > 10 is evidence against weak instrument bias, and filtering out instruments which did not surpass this threshold… |
|  | b) | Report any additional statistics (e.g., assessments of heterogeneity across genetic variants, such as *I^2^*, Q statistic or E-value) | - |  |
| 13 | **Sensitivity analyses and additional analyses** |  |  |  |
|  | a) | Report any sensitivity analyses to assess the robustness of the main results to violations of the assumptions | 10-12 |  |
|  | b) | Report results from other sensitivity analyses or additional analyses | 10-12 |  |
|  | c) | Report any assessment of direction of causal relationship (e.g., bidirectional MR) | - |  |
|  | d) | When relevant, report and compare with estimates from non-MR analyses | - |  |
|  | e) | Consider additional plots to visualize results (e.g., leave-one-out analyses) | - |  |
|  | **DISCUSSION** |  |  |  |
| 14 | **Key results** | Summarize key results with reference to study objectives | 16 | In this study, we used a combined MR-colocalisation framework to estimate the causal effect of genetically proxied gene expression, alternative splicing and protein abundance levels on genetic liability of glioma risk. We found robust evidence that causally implicated 16 molecular traits with glioma risk: ten gene expression evidence levels and six alternative splicing events (one gene shared both expression and splicing causal effects on glioma). |
| 15 | **Limitations** | Discuss limitations of the study, taking into account the validity of the IV assumptions, other sources of potential bias, and imprecision. Discuss both direction and magnitude of any potential bias and any efforts to address them | 18 | Despite using relatively large datasets, our analyses are still likely to suffer from limited statistical power due to restricted sample sizes, particularly in the sQTL dataset (n = 114 to 209). This might lead to some important causal effects not being identified. All three QTL datasets were derived from a mixture of case and control samples. However, the cases were derived from individuals whose disease (e.g. Alzheimer’s disease in BrainQTL) have no known link to glioma risk.  Additionally, as we used the GTEx sGenes, only the top splice event per gene was measured, and therefore there were many splice events which were not tested in our MR analysis.  Most of the MR analyses used a single SNP instrument, which restricts the type of sensitivity analyses that could be performed; however, this is a common phenomenon observed when conducting MR with molecular traits.  SNPs can act via multiple molecular QTL pathways and are not mutually exclusive; SNPs affecting gene expression can also be associated with alternative gene splicing of the same genes. As eQTL and sQTL are both measured by quantifying mRNA levels, this was not unexpected. These SNPs may be legitimately affecting both gene expression and alternative splicing, however if a particular splice variant alters the ability of a gene to be efficiently measured, it appears that gene expression is altered, and the effect is driven by the splicing event. Furthermore, some probes for mRNA will detect commonly splice variants as canonical transcripts, which are included in ‘bulk tissue’ eQTL analysis. This can lead to the false assumption that both expression and splicing events are driving the causal effect, when this is not the case. |
| 16 | **Interpretation** |  |  |  |
|  | a) | Meaning: Give a cautious overall interpretation of results in the context of their limitations and in comparison with other studies | 16,17 | In this study, we used a combined MR-colocalisation framework to estimate the causal effect of genetically proxied gene expression, alternative splicing and protein abundance levels on genetic liability of glioma risk. We found robust evidence that causally implicated 16 molecular traits with glioma risk: ten gene expression evidence levels and six alternative splicing events (one gene shared both expression and splicing causal effects on glioma). Assessing different ‘omics’ data sources can be beneficial to understand the development of complex traits and for drug target-related analyses; each QTL provides an insight into different molecular processes.  For 11 of the genes that we found robust evidence for here, the loci have been previously implicated in GWAS/TWAS of glioma risk. However, these studies did not establish causality of all these genes, as we have. Additionally, we found four genes that not been implicated in previous genetic studies: CEP192 (18p11.21), FAIM (3q22.3), HBEGF (5q13.3) and SLC8A1 (2p22.1). |
|  | b) | Mechanism: Discuss underlying biological mechanisms that could drive a potential causal relationship between the investigated exposure and the outcome, and whether the gene-environment equivalence assumption is reasonable. Use causal language carefully, clarifying that IV estimates may provide causal effects only under certain assumptions | 16,17 | RTEL1-associated sQTLs were associated with an exon-skipping event (GTEx intron ID: 63689132:63689750:clu_27064) present only in RTEL1 transcript ENST00000425905 (P = 4.23 x 10-75 to 6.64 x 10-18). Therefore, it may be the case that previous conflicting evidence on the directionality of the effect of RTEL1 expression on glioma risk may be confounded due to non-canonical transcript variants; though, further studies would be required to ascertain if this is the case. Furthermore, we observed that RTEL1 sQTLs rs6063202 and rs2297440, were found to be strongly associated to glioblastoma (P = 1.00 x 10-13 and 4.00 x 10-46, respectively) and with allergy-related traits (P = 4.78 x 10-8 to 7.84 x 10-19, Supplementary Table S2). These results beget further study and could help to understand the links between allergies, glioma risk and RTEL1. |
|  | c) | Clinical relevance: Discuss whether the results have clinical or public policy relevance, and to what extent they inform effect sizes of possible interventions | - |  |
| 17 | **Generalizability** | Discuss the generalizability of the study results (a) to other populations, (b) across other exposure periods/timings, and (c) across other levels of exposure | 19 | Our analysis is limited to individuals with European ancestry; therefore, it will be important to extend these analyses to individuals of alternative ancestries as such data becomes available. |
|  | **OTHER INFORMATION** |  |  |  |
| 18 | **Funding** | Describe sources of funding and the role of funders in the present study and, if applicable, sources of funding for the databases and original study or studies on which the present study is based | 22 |  |
| 19 | **Data and data sharing** | Provide the data used to perform all analyses or report where and how the data can be accessed, and reference these sources in the article. Provide the statistical code needed to reproduce the results in the article, or report whether the code is publicly accessible and if so, where | 22 |  |
| 20 | **Conflicts of Interest** | All authors should declare all potential conflicts of interest | 22 |  |

This checklist is copyrighted by the Equator Network under the Creative Commons Attribution 3.0 Unported (CC BY 3.0) license.
